# Pre-mRNA Splicing Is a Determinant of Nucleosome Organization

**DOI:** 10.1371/journal.pone.0053506

**Published:** 2013-01-10

**Authors:** Hadas Keren-Shaul, Galit Lev-Maor, Gil Ast

**Affiliations:** Department of Human Molecular Genetics and Biochemistry, Sackler Faculty of Medicine, Tel Aviv University, Tel Aviv, Israel; International Centre for Genetic Engineering and Biotechnology, Italy

## Abstract

Chromatin organization affects alternative splicing and previous studies have shown that exons have increased nucleosome occupancy compared with their flanking introns. To determine whether alternative splicing affects chromatin organization we developed a system in which the alternative splicing pattern switched from inclusion to skipping as a function of time. Changes in nucleosome occupancy were correlated with the change in the splicing pattern. Surprisingly, strengthening of the 5′ splice site or strengthening the base pairing of U1 snRNA with an internal exon abrogated the skipping of the internal exons and also affected chromatin organization. Over-expression of splicing regulatory proteins also affected the splicing pattern and changed nucleosome occupancy. A specific splicing inhibitor was used to show that splicing impacts nucleosome organization endogenously. The effect of splicing on the chromatin required a functional U1 snRNA base pairing with the 5′ splice site, but U1 pairing was not essential for U1 snRNA enhancement of transcription. Overall, these results suggest that splicing can affect chromatin organization.

## Introduction

Alternative splicing (AS) enhances transcriptome and proteome diversity, particularly in mammals, and recent analyses estimate that over 95% of human multi-exon genes produce alternatively spliced transcripts [1]. Until recently, whether an exon was alternatively or constitutively spliced was believed to be solely influenced by sequences in the pre-mRNA, such as those defining exon/intron boundaries, and by binding of splicing regulatory proteins [2]. It has now become apparent, however, that transcription by RNA polymerase II (RNAPII) and chromatin structure contribute to alternative splicing regulation [3], [4].

Nucleosome positioning may be linked with exon-intron architecture and splicing through mechanisms involving transcription by RNAPII [5]–[9]. Evidence for a reciprocal coupling between transcription and splicing is strong [5], [10]–[13]. A high percentage of splicing events occur co-transcriptionally, meaning that introns are removed from the pre-mRNA while the nascent transcript is still tethered to the DNA by RNAPII [11]. U1 snRNP is associated with RNAPII, and this interaction is involved in coupling of different processes related to gene expression [14]–[17]. Spliceosome assembly is linked to RNAPII pausing [18], and splicing was found to affect transcription initiation [19], [20], elongation [21], and termination [18].

Chromatin structure and epigenetic markers also influence the recognition of exons and splice site choice: At the DNA level, exons have increased nucleosome occupancy levels compared with the flanking intron sequences, and the histones in nucleosomes bound to exons are enriched in certain modifications [3], [4]. Changes in chromatin structure, caused by histone modifications or DNA methylation, affect splicing. For instance, histone deacetylase (HDAC) activity can modulate AS [22], treatment with HDAC inhibitors affects AS of the extra domain I (EDI) exon in fibronectin [23], and siRNA targeting of this exon increases epigenetic marks and reduces the RNAPII elongation rate as well as levels of exon inclusion [24]. Also, membrane depolarization of neuronal cells triggered hyperacetylation surrounding exon 18 of the neural cell adhesion molecule (NCAM), increased transcriptional elongation, and resulted in skipping of this exon [25]. Furthermore, increased levels of the transcriptional repressor HP1γ and elevated levels of the epigenetic mark H3K9me3 are associated with inclusion of alternative exons [26], and the zinc finger DNA-binding protein CTCF causes local RNAPII pausing and influences exon inclusion [27]. Finally, chromatin remodelers (e.g., SWI/SNF complex) regulate both splicing and RNAPII elongation rate [28]–[30].

Not only can chromatin organization affect transcription, but transcription can potentially affect chromatin organization. For instance, perturbation of the phosphorylation status of RNAPII alters epigenetic marking by regulating H3 methylation [5], [31]. RNAPII also plays a role in nucleosome rearrangement at promoters and in the decrease in nucleosome occupancy during transcription within gene bodies. Loss of RNAPII results in relaxation of a chromatin structure [32], and transcription was shown to induce nucleosome organization in vivo [32]–[34].

In this study, we set out to examine whether AS can influence chromatin organization. We examined the effect of splicing on nucleosome organization using a minigene system that exhibits a change in the AS pattern as a function of time. We found clear evidence that splicing affected nucleosome organization. We further found that this effect was present endogenously. The effect of splicing on the chromatin required a functional U1 snRNA base pairing with the 5′ splice site (5′ss); furthermore, this effect was probably independent of RNAPII transcription. This implies a very interesting interplay among chromatin organization and AS.

## Results

**Figure 1:**
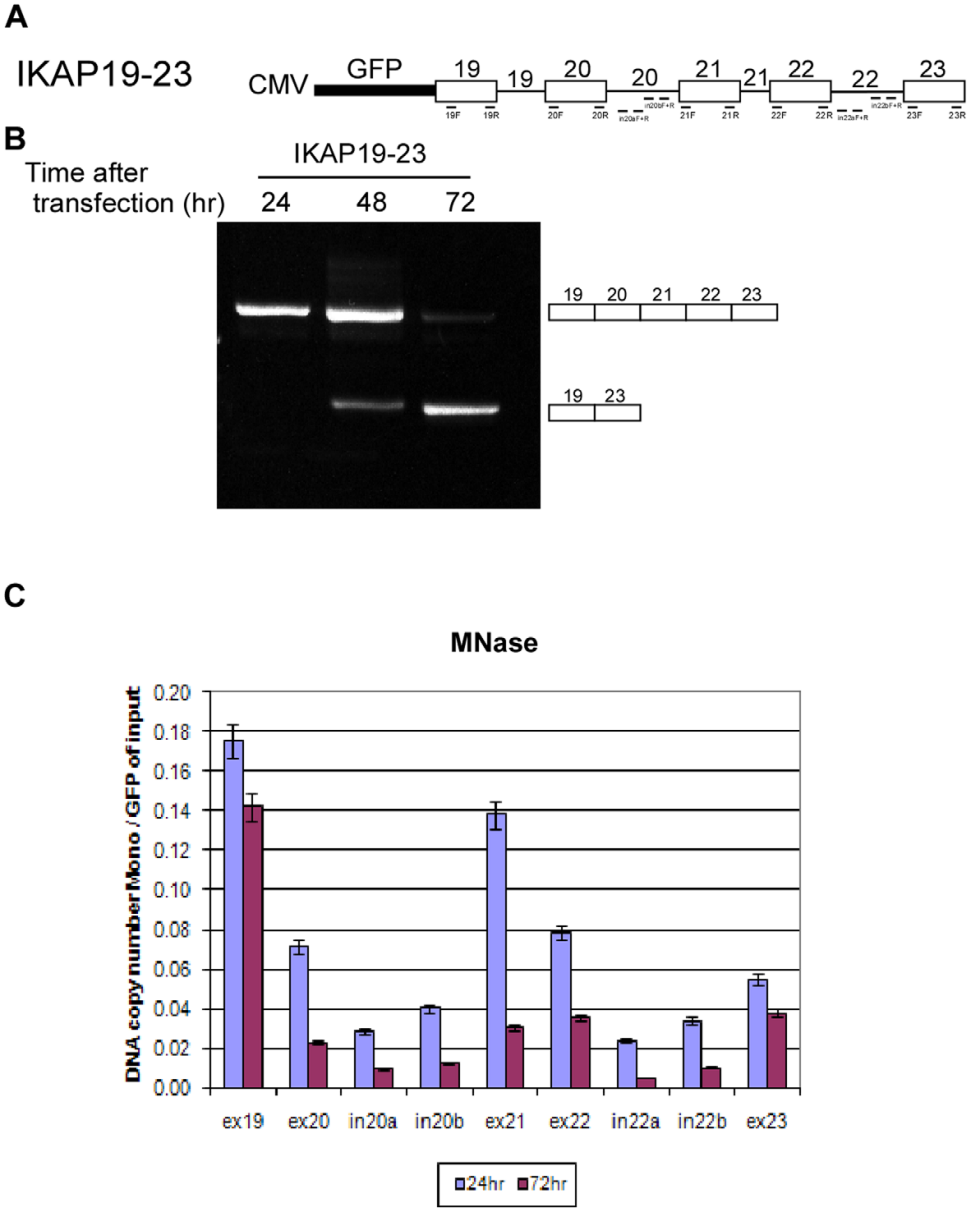
The shift in alternative splicing is linked to chromatin organization. (**A**) Schematic diagram illustrating the exons (boxes) and introns (lines) in the IKAP19–23 minigene, which was inserted into pEGFP-C3 plasmid. The location of the different primers used is indicated. Drawing is not to scale. (**B**) The IKAP19–23 minigene was transfected into 293 cells. RNA samples were extracted 24, 48, or 72 hr following transfection. The splicing products were separated on a 2% agarose gel after RT-PCR reaction using appropriate primers. The PCR products were eluted and sequenced. (**C**) IKAP19–23 minigene was transfected into 293 cells. Nuclei were extracted 24 and 72 hr following transfection. Half of each sample was treated with MNase and half was untreated. Mononucleosomal DNA was extracted from an agarose gel and subjected to absolute QPCR analysis with primers that cover most of the minigene. Data are presented as DNA copy number and were normalized to transfection efficiency using primers for the GFP area of the plasmid using untreated samples.

In order to investigate the connection between chromatin organization and AS, we developed a minigene system that exhibits a shift in splicing pattern in cells as a function of time following transfection. The minigene is called IKAP19–23 and contains five exons of the *IKBKAP* gene and the introns in between them in a pEGFP-C3 vector (Fig. 1A). The minigene was transfected into 293 cells, RNA was extracted 24, 48, or 72 hr following the transfection, and the splicing products were amplified. At 24 hr after transfection, there was full inclusion of all exons of the minigene; however, after 72 hr the inclusion isoform disappeared and a skipped isoform, displaying ligation of exon 19 to exon 23, appeared (Fig. 1B). Thus, as a function of time, the splicing pattern changed from inclusion to skipping of the internal exons. The shift in splicing as a function of time was unique to the IKAP19–23 minigene: Other minigenes analyzed showed constitutive splicing at all times tested (Fig. S1A, D). It should be mentioned that a point mutation at the 5′ss of intron 20 of the *IKBKAP* gene leads to Familial Dysautonomia, an autosomal recessive congenital neuropathy [35]; however, the minigene depicted here is the wt form of the gene. Also, the unique change in splicing as a function of time does not exist in the endogenous gene.

The recently discovered increased nucleosome occupancy in exons compared with their flanking introns [36], together with the effect of chromatin organization on AS [4], [5], prompted us to correlate the changes observed in the splicing pattern of the minigene with nucleosome occupancy. Hence, we evaluated whether the changes in splicing pattern were reflected by changes in nucleosome occupancy. Transfected plasmids form a nucleosomal chromatin structure that can be detected using an MNase digestion method [37]–[40]. We transfected the minigene into 293 cells and extracted the nuclei 24 hr and 72 hr after transfection. The nuclei were treated with MNase and DNA fragments of ∼150 nucleotides (mononucleosomal DNA) were isolated from an agarose gel. The DNA fragments were subjected to absolute real-time quantitative PCR (QPCR) analysis using primer pairs that covered most of the minigene. In order to correct for differences in transfection levels, the results were normalized against the plasmid's GFP amplicon area from undigested nuclei. The differences in nucleosome occupancy were found to be statistically significant by *t*-test analysis. There was lower nucleosome occupancy (expressed as DNA copy number) at 72 hr compared to 24 hr after the transfection (Fig. 1C), indicating a more relaxed chromatin structure after 72 hr. The decrease in nucleosome occupancy was observed throughout the minigene and was correlated with the splicing pattern change from inclusion to skipping.

The exons had higher nucleosome occupancy than did flanking intronic sequences, consistent with previous findings [36], [41], [42]. In addition, higher nucleosome occupancy was observed toward the 5′ end of the minigene than the 3′ regions, similar to data reported previously [43], [44]. These results imply that the minigene system faithfully mimicked the endogenous state of nucleosome occupancy. These results were also verified by performing chromatin immunoprecipitation (ChIP) with a histone H3 antibody (H3-ChIP): Lower levels of H3 precipitation were observed at 72 hr than at 24 hr after the transfection (Fig. S2A), thus the ChIP results support the MNase results. The differences in ChIP values were found to be statistically significant by *t*-test analysis. Furthermore, chromatin was more accessible to DNase I at 72 hr than at 24 hr following the transfection (Fig. S2B), which confirms that our results obtained 72 hr after the transfection were not due to incomplete digestion by the MNase because of DNA compaction. These data show that the chromatin structure of the plasmid is more open at later than at earlier time points.

In the minigenes that were constitutively spliced at both 24 and 72 hr (Fig. S1A, D), nucleosome occupancy increased between 24 to 72 hr as shown by MNase treatment and H3-ChIP analysis (Fig. S1B, C, E, and F). This increase was opposite the decrease observed with the IKAP19–23 minigene (Fig. 1C and S2A), which has a shift in the splicing pattern (Fig. 1B). These observations indicate that, in our minigene model system, the splicing pattern correlates with the organization of the chromatin.

It has been shown previously that chromatin structure can affect AS (see Introduction). This was also validated in our system using trichostatin A (TSA) or sodium butyrate (NaB), which inhibit HDACs and cause relaxation of the chromatin [45], [46] (Fig. S3A), and campthothecin (CPT), which inhibits topoisomerase I (Top1), and should prevent the release of DNA supercoiling during transcription [14], [47] (Fig. S3B). These results are not consistent with previous results in fibronectin and NCAM minigene systems [23], [25]; in these systems, TSA treatment led to skipping. This might be explained by the differences in the gene sequences used in each study, since the activity of HDAC has been shown to modulate AS in different ways [22].

**Figure 2:**
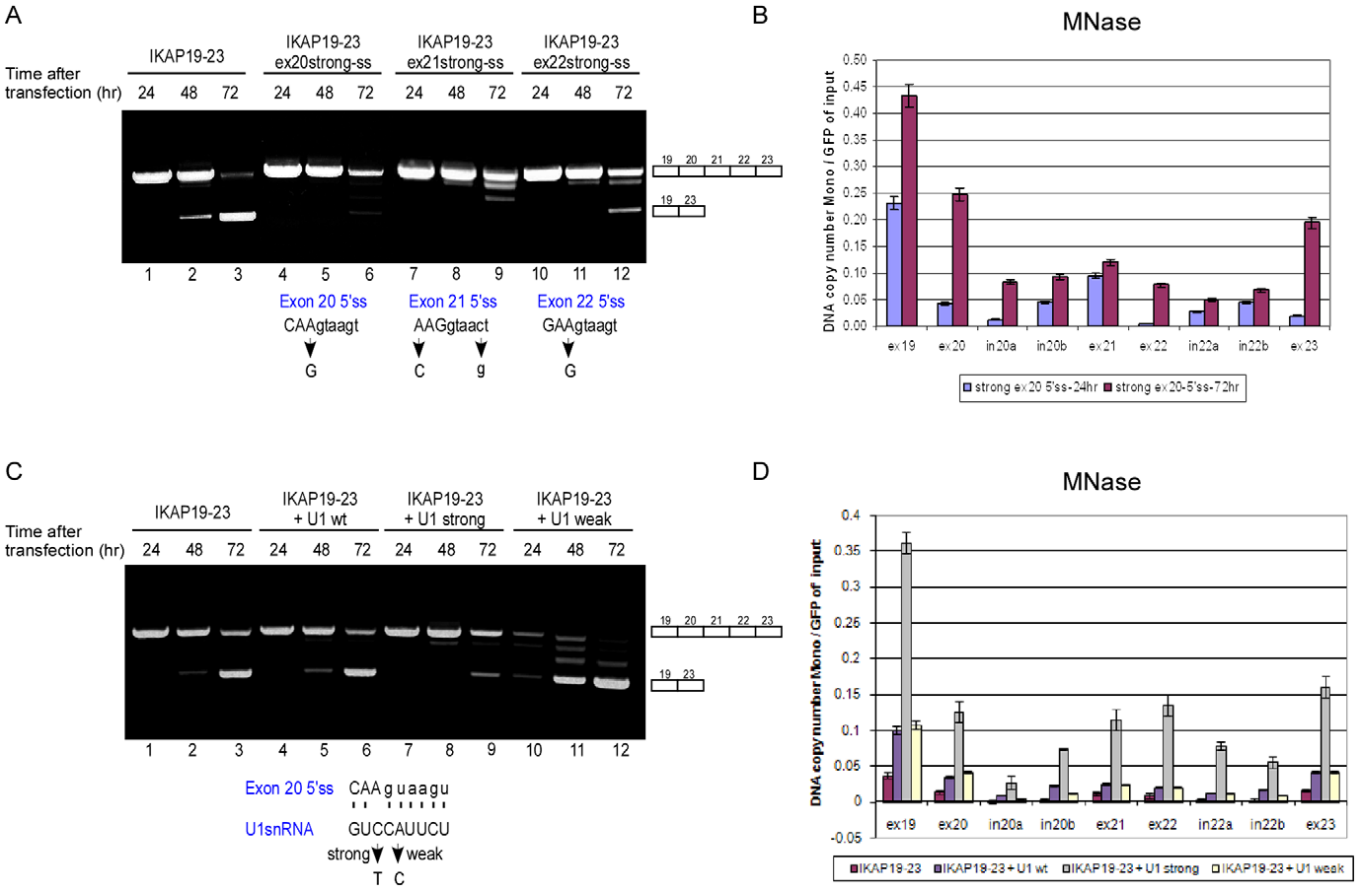
Alternative splicing affects nucleosome occupancy. (**A**) IKAP19–23 minigenes with different mutations that strengthened the 5′ss of each internal exon were transfected into 293 cells and splicing was evaluated. “Strong-ss” indicates a mutation that strengthened the splice site score [1]. The positions of the 5′ss mutations for each minigene are indicated with an arrow in the lower part of the panel. (**B**) The IKAP19–23 minigene with a strong exon 20 5′ss was transfected into 293 cells, and nuclei were extracted 24 and 72 hr following transfection. Half of each sample was treated with MNase and half was untreated. Mononucleosomal DNA was extracted from an agarose gel and subjected to absolute QPCR analysis with primers that cover most of the minigene. Data are presented as DNA copy number and were normalized to transfection efficiency using primers for the GFP area of the plasmid using untreated samples. (**C**) Co-transfection of the IKAP19–23 minigene into 293 cells was performed with plasmids that express U1 snRNAs. Three different U1 snRNA plasmids were used (as shown in the lower part of the panel): wt U1, U1 with a mutation that strengthens its base pairing with exon 20 5′ss (U1 strong), and U1 with a mutation that weakens the base pairing with exon 20 5′ss (U1 weak). Watson-Crick base pairing is marked by dashed line. The position of the relevant mutation in U1 snRNA is indicated with an arrow. RNA samples were extracted 24, 48, or 72 hr following transfection. The splicing products were separated on a 2% agarose gel after RT-PCR. The PCR products were eluted and sequenced. (**D**) The IKAP19–23 minigene was co-transfected into 293 cells with either U1 wt, U1 strong or U1 weak plasmid. 72 hr after the transfection, nucleosome occupancy was determined using an MNase assay as above. All experiments were repeated independently three times, and the results shown are representative of an average experiment. QPCR experiments were performed in triplicate; results shown are mean values ± SD.

Since chromatin organization does affect AS, we questioned whether AS affected chromatin organization as well. As the selection of exons is influenced by the splice site strength [48], we tested the effect of the splice site sequence on the time-dependent shift in splicing. We strengthened the 5′ss of each internal exon by site-directed mutagenesis. Remarkably, strengthening of the 5′ss of one internal exon was sufficient to significantly reverse the shift from mostly exon skipping to mostly exon inclusion (Fig. 2A, compare lanes 3, 6, 9 and 12). For example, there was a 3.5-fold increase in inclusion with the strong exon 20 5′ss compared to the wild-type (wt) IKAP19–23 as measured by QPCR (Fig. S4A). When we analyzed nucleosome occupancy in the minigene containing a stronger 5′ss in exon 20 by MNase and H3-ChIP analyses, we observed an increase throughout the minigene in nucleosome occupancy at 72 hr following transfection compared to 24 hr (Fig. 2B and S5A). Over this time frame, we observed a decrease in nucleosome occupancy of the wt minigene (Fig. 1C). These results imply that, in our minigene model system, AS can affect nucleosome occupancy: Inclusion of exons is correlated with an increase in nucleosome occupancy and skipping of exons is correlated with a decrease in nucleosome occupancy.

As these results were obtained when the DNA was mutated, we co-transfected the IKAP19–23 minigene with a U1 snRNA plasmid containing a compensatory mutation that strengthens U1 snRNA base pairing with the wt 5′ss of exon 20. Similar to the effect of strengthening the exon 20 5′ss, the co-transfection with the mutant U1 almost completely abrogated the shift in the splicing pattern as a function of time (Fig. 2C, compare lane 3 to 9). A mutation in the U1 snRNA plasmid that weakened the base pairing of U1 with the 5′ss had the opposite effect; it caused the shift to appear even earlier (Fig. 2C, compare lane 2 to 11). The ratio of inclusion to skipping with transfection of the different U1 plasmids is presented in Figure S4B. 72 hr following the transfection, both MNase and H3-ChIP analyses showed higher nucleosome occupancy throughout the minigene when a mutant U1 was used to restore exon inclusion; co-transfection of either a wt U1 or a mutant U1 that weakened base pairing with the 5′ss of exon 20 and led to earlier exon skipping resulted in a substantially lower increase in nucleosome occupancy than that of co-transfection with U1 strong (Fig. 2D and S5B). The slight increase in nucleosome occupancy with the U1 wt or U1 weak plasmids compared to control might be due to the increase of U1 concentration in the cells. This might disrupt the equilibrium of the endogenous U1 snRNP and may affect chromatin conformation. For example, U1 can interact with RNAPII and directly with chromatin via transcription factor TATA box-binding protein (TBP) - associated factor 15 (TAF15) [49]–[51].

We then co-transfected the IKAP19–23 minigene with plasmids that express splicing regulatory proteins. Among the splicing factors examined, SRSF2 led to skipping of exon 20 and SRSF1 led to skipping of exon 20 and 21; hnRNP A1 did not affect the splicing pattern of the minigene (Fig. S6A). SRSF2 and SRSF1 resulted in a decrease in nucleosome occupancy, whereas hnRNP A1 led to an increase in nucleosome occupancy of the plasmid (Fig. S6B). Inhibiting splicing using meayamycin, an analog of spliceostatin A that binds to SF3b and promotes pre-mRNA accumulation [52]–[54], resulted in skipping of the internal exons (Fig. S7A) and lower nucleosome occupancy (Fig. S7B).

**Figure 3:**
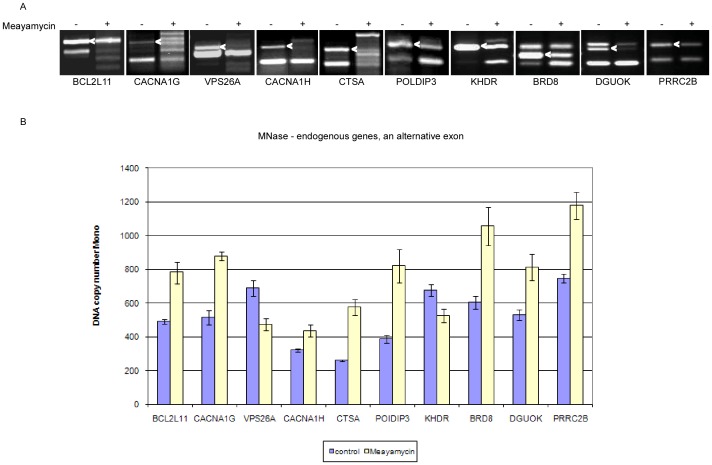
Alternative splicing affects nucleosome occupancy in endogenous genes. (**A**) HeLa cells were treated with 10 nM meayamycin and RNA was extracted 24 hr later. Splicing products were separated on a 1.5% agarose gel after RT-PCR reaction using appropriate primers for the flanking the alternative exon (marked with an arrow). Ten endogenous genes were analyzed: BCL2-like 11 (apoptosis facilitator; BCL2L11); calcium channel, voltage-dependent, T type, alpha 1G subunit (CACNA1G); vacuolar protein sorting 26 homolog A (*S. pombe*) (VPS26A); calcium channel, voltage-dependent, T type, alpha 1H subunit (CACNA1H); cathepsin A (CTSA); polymerase delta interacting protein 3 (POLDIP3); KH domain containing, RNA binding, signal transduction associated 1 (KHDR); bromodomain containing 8 (BRD8); deoxyguanosine kinase, nuclear gene encoding mitochondrial protein (DGUOK); and proline-rich coiled coil 2B (PRRC2B). (**B**) DNA was extracted from HeLa nuclei 24 hr after meayamycin treatments. An MNase assay was then performed and the mononucleosomal DNA was subjected to absolute QPCR analysis on the alternative exon. Data are presented as DNA copy number.

In order to obtain a broader perspective, we set out to examine whether splicing impacts chromatin organization endogenously. We selected ten genes, *BCL2L11*, *CACNA1G*, *CACNA1H*, *VPS26A*, *CTSA*, *POLDIP3*, *KHDR*, *BRD8*, *DGUOK*, and *PRRC2B*, that show changes in the splicing patterns under different conditions [22] [55]. We treated HeLa cells with meayamycin and examined the AS pattern before and after the treatment by RT-PCR (Fig. 3A). We then evaluated nucleosome occupancy of the alternative exon of each gene (Fig. 3B). For the *BCL2L11* and *CACNA1G* genes, meayamycin treatment resulted in reduction of the skipping isoform (and a slight increase in the inclusion isoform), and increased nucleosome occupancy of the alternative exon (marked with an arrow in Fig. 3B). For *VPS26A*, *CACNA1H*, *POLDIP3*, *KHDR*, *BRD8*, *DGUOK*, and *PRRC2B* meayamycin reduced levels of the inclusion isoform. A decrease in nucleosome occupancy was observed for two of the genes (*VPS26A* and *KHDR*) and an increase in nucleosome occupancy for the rest. Meayamycin caused a general disruption in splicing machinery for *CTSA*, similar to that reported previously [56], which in turn resulted in elevated nucleosome occupancy. Our results indicate that a change in the splicing pattern leads to a change in nucleosome occupancy in cells, confirming our hypothesis based on data from the minigene system that splicing can affect chromatin organization.

**Figure 4:**
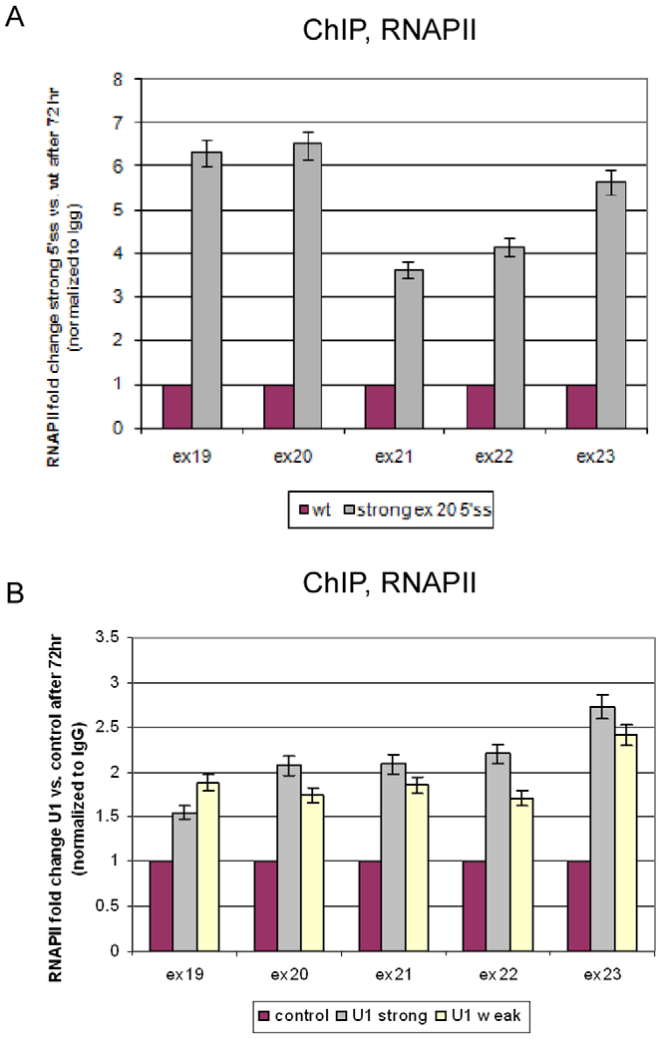
Splicing affects RNAPII occupancy. (**A**) At 72 hr following transfection with either the wt or strong exon 20 5′ss minigene, cells were collected and used for an RNAPII-ChIP analysis. The precipitated DNA fragments were subjected to QPCR. Enrichment values were normalized to the unbound fraction, to a non-specific IgG antibody, and to the GFP area of the plasmid. Results are presented as RNAPII fold change between strong exon 20 5′ss and the wt plasmid. (**B**) At 72 h following co-transfection of IKAP19–23 and U1 plasmids, RNAPII-ChIP was performed. All experiments were repeated independently three times, and the results shown are representative of an average experiment. QPCR experiments were amplified in triplicate; results shown are mean values ± SD.

Since AS and transcription are coupled processes, we set to examine the involvement of RNAPII in the effect of splicing on chromatin organization. A ChIP analysis using an RNAPII antibody (RNAPII-ChIP), as described previously [57], of the wt minigene revealed a decrease in RNAPII occupancy at 72 hr compared to 24 hr (Fig. S8A). This decrease suggests that a higher elongation rate of RNAPII is correlated with exon skipping; this was confirmed by an RNAPII processivity analysis (Fig. S8B). The strong exon 20 5′ss mutant plasmid displayed an increase in RNAPII occupancy between 24 hr and 72 hr (data not shown). Treatment of transfected cells with 5,6-dichloro-1-β-D-ribofuranosyl-benzimidazole (DRB), which inhibits elongation by RNAPII, restored the inclusion of the internal exons as a function of the concentration used (Fig. S8C), and increased nucleosome occupancy throughout the minigene (Fig. S8D). The effect of DRB on splicing is different than that of CPT, which was also reported to inhibit RNAPII [57], [58]. This is because CPT may cause either skipping [59] or inclusion of exons [60], and inhibition of transcription can affect recognition of exons differently [57]. We further compared RNAPII distribution between two minigenes; one contains the wt 5′ss (and displayed mostly skipping of exons) and the other contains a mutation that strengthens exon 20 5′ss (and exons were mostly included). RNAPII occupancy was examined 72 hr following transfection and was higher for the mutant compared to the wt minigene (Fig. 4A). When the splicing pattern was altered to skipping by meayamycin treatment, RNAPII occupancy was decreased (Fig. S9). Transfection of the U1 snRNA plasmids also resulted in an increase in RNAPII occupancy (Fig. 4B); surprisingly, this increase was similar for both U1 strong and U1 weak. These findings indicate that U1 snRNA enhances transcription regardless of its ability to base pair with the 5′ss. For the effect of splicing on the chromatin, however, a functional U1 base pairing with the 5′ss was required. These findings suggest that AS affects chromatin organization through a mechanism that does not appear to depend on the elongation rate of RNAPII.

Interestingly, in the examined endogenous genes there was a correlation between the effect of meayamycin on nucleosome occupancy and RNAPII occupancy: An increase in RNAPII occupancy following meayamycin treatment was observed when nucleosome occupancy was increased between treated and untreated cells, and a decrease in RNAPII occupancy was observed when nucleosome occupancy decreased between treated and untreated cells (Fig. S10). This strengthens the connection previously suggested between transcription and chromatin organization.

## Discussion

The results reported here provide evidence for a layer of regulation of gene expression that involves bi-directional interplay between the processes that act on DNA and RNA in the cell. Our data indicate that the regulation of AS at the RNA level influences an upstream process – the organization of the chromatin. U1 snRNA binding to the 5′ss provides the necessary signal from the splicing reaction back to chromatin organization.

Several potential mechanisms might link chromatin structure to the processes of transcription and splicing. It may be that a nucleosome bound to an exon acts as a “speed bump”, slowing RNAPII elongation and leading to an increase in the inclusion level of that exon. Another possibility is that the “preferred” nucleosome positioning along the exon marks it with specific histone modifications that lead to interactions with the splicing machinery, allowing more efficient recognition of the exon [36], [41]. Alternatively, the CTD domain of RNAPII may serve as a landing pad for splicing factors that also directly bind chromatin. The CTD of RNAPII may also interact with chromatin remodelers that alter chromatin conformation along the exons and thereby recruit splicing factors to exonic regions [3], [9], [61], [62].

Even though the effect of chromatin organization on AS is unquestionable, little is known about the effect that AS has on chromatin organization. We found that skipping of the internal exons depended on the strength of the splice site – a stronger splice site or the presence of a mutated U1 snRNA with better binding to one of the internal exons restored the inclusion of exons. Remarkably, altering splice site strength or strengthening U1 snRNA base pairing with the 5′ss reversed the changes in nucleosome occupancy (Fig. 2). We further showed that transfecting different splicing proteins (Fig. S6) or inhibiting splicing with meayamycin changed the splicing pattern and nucleosome occupancy (Fig. S7). These findings suggest that AS indeed has an impact on chromatin organization.

We first demonstrated an effect of splicing on chromatin organization by generating a very elegant minigene model system. We further extend our analysis endogenously to test the effect on chromatin organization using a specific splicing inhibitor (Fig. 3). This drug had affected the splicing pattern in all our selected genes and nucleosome occupancy was altered as well. RNAPII occupancy was correlated with the changes in nucleosome occupancy (Fig. S10). In the minigene system, inclusion was correlated with higher nucleosome occupancy, which was not always the case in the endogenous analysis. This is not surprising since endogenously the splicing reaction depends on a variety of factors, such as the genomic surrounding. Our data are consistent with the link between AS and the elongation rate of RNAPII, which was also first demonstrated using a minigene system from the EDI gene [10], and only subsequently shown in a genome-wide manner. Although the EDI minigene system showed an increase in the inclusion level of exons when a slow RNAPII was used, the genome-wide analysis demonstrated that only a fraction of alternative exons changed their inclusion level due to a change in the elongation rate of RNAPII. In those that were altered, some showed an increase and some displayed a decrease in the inclusion level [57].

How can the splicing process affect the organization of the chromatin? Chromatin organization affects transcription, which is reciprocally coupled to splicing, and transcription can potentially affect chromatin organization. This regulation might be affected through changes in the elongation rate of RNAPII or by recruitment of factors as both are known to influence spliceosome assembly. In our minigene model system, we observed that an increase in nucleosome occupancy correlated with inclusion of exons. This supports the “speed bump” model in which the nucleosome acts as a fluctuating barrier to RNAPII, slowing down transcription and enabling access by the splicing machinery to the exon. This is similar to the effect of inhibitors of transcription that shifted the splicing pattern to exon inclusion (Fig. S8C) and caused increased nucleosome occupancy (Fig. S8D). These results are consistent with those of another study that showed that transcription inhibition increased nucleosome occupancy [63].

The decrease in nucleosome occupancy observed in our system at 72 hr following transfection correlated with the skipping of exons. We found that RNAPII has an accelerated elongation rate at the latter time point (Fig. S8A, B), which supports the kinetic model in which splicing is correlated with the time lag between transcription of one exon and the next [7], [10]. However, we must keep in mind that nucleosomes spontaneously undergo conformational changes [64] and that the polymerase does not actively separate DNA from histones but rather rectifies nucleosomal fluctuations [65]. Chromatin remodeling factors such as the SWI-SNF complex may increase the frequency or extent of these fluctuations [63]. Specific splicing factors transmit epigenetic information between the nucleosome and the splicing machinery. For example, the H3K4me3 binding protein CHD recruits U2 snRNP [66], and the H3K36me3 binding protein MRG15 interacts with the splicing factor PTB [67]. Also, Hu proteins that participate in splicing regulation can induce local histone acetylation in regions surrounding alternative exons [68], and splicing proteins such as serine/arginine proteins (SR) interact with the chromatin [6], [67], [69]. Very recently, splicing was found to be necessary for establishing normal patterns of H3K36 trimethylation [70], and splicing enhanced H3K36me3 histone marking by promoting the recruitment of H3K36 methyltransferase HYPB/Setd2 to the elongating RNAPII [71].

U1 snRNA has been shown to stimulate transcription [20], [50], [51], and human U1 snRNA was found to be associated with the transcription factor TAF15 [49]. We found that a strong base pairing with the 5′ss increased RNAPII occupancy (Fig. 4A). Over-expression of U1 snRNA also increased RNAPII occupancy; but, this was regardless of whether the exogenous U1 had the ability to base pair with the 5′ss (Fig. 4B). A functional base pairing between U1 and the 5′ss was required for effects on chromatin structure (Fig. 2B), however, which might indicate that the effect of AS on chromatin organization is not fully dependent on the elongation rate of RNAPII. U1 snRNA is part of the spliceosome, which is composed of five snRNPs and additional proteins. U1 associates with the 5′ss to initiate the process of splicing. The abundance of U1 snRNA in human cells far exceeds that of the other snRNAs [72]. This may indicate that U1 snRNA has roles in the cell in addition to splicing initiation. Indeed, Kaida *et al.* found a splicing-independent function for U1 snRNP in protecting pre-mRNAs from premature cleavage and polyadenylation [17]. The base pairing of U1 to the pre-mRNA was required for this action. It is therefore tempting to consider the base pairing of U1 to the 5′ss as a potential factor that regulates the effect of splicing on chromatin organization.

In this work we demonstrated that splicing can affect nucleosome organization, suggesting a bi-directional interplay between chromatin organization and splicing. The nucleosome positioning in exons seems to encourage the proper location of molecular interactions across the exon, contributing to exon definition. Our data reveal another level of complexity in eukaryotic splicing mechanism and add another piece to the puzzle of AS regulation. Our demonstration of the effect of AS at the RNA level on organization of the chromatin at the DNA level is the preface for further research on this regulation mechanism.

## Methods

### Plasmid construction

The desired minigenes were generated by amplifying a human genomic fragment using PCR with a PfuTurbo DNA polymerase (Stratagene). Each primer contained an additional sequence encoding a restriction enzyme. The PCR products were restriction digested and inserted into the pEGFP-C3 plasmid (Clontech). All constructs were sequenced. The IKAP19–23 minigene was from the *IKBKAP* gene (inhibitor of kappa light polypeptide gene enhancer in B-cells, kinase complex-associated protein) containing exons 19 through 23. The IKAP19–21 minigene contains exons 19 through 21 of the *IKBKAP* gene. The IMP minigene contains exons 11 through 13 from the *IMP* gene (IGF-II mRNA-binding protein). The wt U1 snRNA plasmid was provided by A. Weiner. The SRSF1, SRSF2, and hnRNP A1 plasmids were provided by A. Krainer and S. Stamm.

### Site-directed mutagenesis

The 5′ splice site mutations in internal exons of the IKAP19–23 minigenes and the mutations in the U1 snRNA plasmids were incorporated by site-directed mutagenesis. Specific overlapping oligonucleotide primers that contained the desired mutation were used to insert the mutation using PfuTurbo DNA polymerase (Stratagene). After PCR amplification, the reaction was digested with DpnI restriction enzyme (New England BioLabs) for 1 hr at 37°C; 1–3 µL of the reaction were transformed into *E. coli* XL-1. Colonies were picked for mini-prep extraction (Qiagen) and subsequently sequenced.

### Cell culture, treatment, and transfection

293 cell lines were cultured in Dulbecco's modified Eagle's medium (DMEM) with 10% fetal calf serum (FCS), 0.29 mg ml^−1^ L-glutamine, 100 U ml^−1^ penicillin, and 0.1 mg ml^−1^ streptomycin at 37°C in a humidified atmosphere with 5% CO_2_. The cells were seeded one day prior to transfection and reached about 60% confluence. Plasmids were transfected into the cells using TransIT-LT1 Reagent (Mirus) according to the manufacturer's protocol. Trichostatin A (TSA), sodium butyrate (NaB), campthothecin (CPT), and 5,6-dichloro-1-β-D-ribofuranosylbenzimidazole (DRB) were purchased from Sigma. Meayamycin was synthesized by Sami Osman and Kazunori Koide from the Department of Chemistry, University of Pittsburgh, Pittsburgh, Pennsylvania.

### RNA isolation and RT-PCR amplification

After the indicated time following the transfection, total RNA was extracted using TRI reagent (Sigma) according to the manufacturer's protocol. RNA concentrations were determined using a NanoDrop ND-1000 spectrophotometer. Total RNA (2 µg) was amplified using avian myeloblastosis virus reverse transcriptase (RT-AMV, Roche) with an oligo(dT) reverse primer. *IKAP* transcripts were amplified with Red Load Taq master mix (Larova) using primers for exon 19 and the multiple cloning site (MCS) of the plasmid. *IMP* transcripts were amplified using primers to exon 11 and the MCS of the plasmid. Sequences will be provided upon request. The splicing products were separated on 2% agarose gels and were sequenced. All RT-PCR experiments were performed independently three times. The average inclusion (exon 19, exon 20, exon 21, exon 22 and exon 23) to skipping (exon 19 and exon 23) ratios were calculated by imageJ and are presented in Table S1.

### Nuclei extraction and MNase digestion

At the indicated time following the transfection, the cells were trypsinized and centrifuged for 5 min at 2000 *g*. After washing with PBS, the pellet was suspended in lysis buffer I (0.3 M sucrose, 60 mM KCL, 15 mM NaCl, 5 M MgCl_2_, 0.1 mM EGTA, 15 mM Tris-HCl, pH 7.5, 0.5 M DTT, 0.2 mM PMSF and protease inhibitor cocktail), precipitated again, and then resuspended in a lysis buffer II containing 0.2% IGEPAL-CA630 (Sigma). After incubation on ice, the lysed cells were added to a 1.2 M sucrose cushion. Nuclei were obtained after centrifugation for 20 min at 10,000 *g* at 4°C. For MNase digestion, the pellet was suspended in MNase digestion buffer (0.32 M sucrose, 50 mM Tris-HCl, pH 7.5, 4 mM MgCl_2_, 1 mM CaCl_2_, 0.5 mM DTT, 0.2 mM PMSF and protease inhibitors cocktail). Half of the nuclei (1×10^6^) were incubated with MNase (Worthington Biochemical Corporation, 10 units/µl) for 10 min at 37°C, and half were untreated. The reaction was stopped by adding 20 mM EDTA. Proteins were digested overnight at 65°C using Proteinase K (New England Biolabs). DNA samples (digested and undigested) were purified by phenol∶chloroform∶isoamylalcohol (25∶24∶1; Sigma) extraction. DNA samples were then treated with DNase-free RNase (Sigma) for 1 hr at 37°C. The digested samples were then run on an agarose gel and the mononucleosomal DNA, a ∼150 bp band, was excised from the gel and purified using the Promega Wizard SV Gel and PCR clean-up system. The mononucleosomal DNA was used as template for QPCR, and the results were normalized to the GFP amplicon area of the undigested DNA as a transfection control.

### DNase I digestion and Southern blot assay

Nuclei were isolated as described above, except that 0.1 mM spermine (Sigma) and 0.25 mM spermidine (Sigma) were added to the lysis buffers. One million nuclei were digested with 20 U DNase I (New England BioLabs) for 15 min at 37°C. The reaction was stopped by adding EDTA to a final concentration of 5 mM following 10 min at 75°C. The digested DNA was treated with DNase-free RNase (Sigma) for 1 hr at 37°C. Proteins were digested overnight at 65°C using Proteinase K (New England BioLabs). The DNA was extracted with an equal volume of phenol∶chloroform∶isoamylalcohol (25∶24∶1; Sigma). In order to estimate plasmid amounts for each sample, clean DNA was subjected to QPCR assay (as described below) using primers to the GFP region. For the Southern blot assay, 15 µg of the plasmid were run on a 1.5% agarose gel and transferred onto Hybond N+ membrane (Amersham) by capillary blot procedure, following hybridization with a DIG-labeled DNA probe (fragment of the GFP area in the pEGFP vector). The membrane was visualized using CDP-Star (Roche) and exposure to X-ray film.

### QPCR analysis

QPCR was performed on the Stratagene Mx3005P System using the Absolute Blue QPCR SYBR Green ROX mix (Thermo Scientific). Analysis was performed using the MxPro 4.01 software. All primer pairs yielded a linear standard curve with an R^2^>0.985 and efficiency of reaction was between 90–105%.

For the nucleosome occupancy analysis of the IKAP19–23 minigene, primer pairs were designed to amplify regions of exons and introns throughout the plasmid. Absolute values were calculated based on a standard curve of the IKAP 19–23 minigene plasmid. The standard curve was created based on calculation of the mass of a single plasmid molecule, following preparation of serial dilutions of the plasmid and QPCR. For specific details on calculation of absolute values see the Applied Biosystem handbook. In order to correct measurements for differences in transfection levels, the results were normalized against the plasmid's GFP amplicon area of the undigested nuclei (GFP of input). Results are presented as DNA copy number of the mononucleosome.

For nucleosome occupancy analysis on endogenous genes, primers were designed to hybridize the alternative exon of each gene. For the standard curve used to calculate the absolute DNA copy number we used genomic DNA; for details see the Applied Biosystem handbook.

For ChIP analysis, primer pairs were designed to hybridize several regions throughout the minigene. Enrichment values were normalized to the unbound fraction, to a non-specific IgG antibody, and to the GFP area of the plasmid. Results are presented as the antibody fold change treatment compared to control. Primers sequences will be provided upon requested.

All experiments were performed three times independently unless indicated otherwise. The differences in nucleosome occupancy or ChIP analysis were found to be statistically significant using *t*-test analysis.

### Chromatin immunoprecipitation

Cells were crosslinked by addition of formaldehyde to a final concentration of 1% for 10 min at room temperature. The crosslinking reaction was stopped by adding glycine to a final concentration of 0.125 M and incubation for 5 min at room temperature. Cells were collected in PBS and centrifuged for 4 min at 931 *g* at 4°C. The pellet was suspended in lysis buffer (10 mM HEPES, pH 6.5, 10 mM EDTA, 0.5 mM EGTA, 0.25% Triton X-100) and incubated on ice for 10 min. Following a 5-min centrifugation at 3724 *g* at 4°C, the pellet was suspended in a second lysis buffer (10 mM HEPES, pH 6.5, 1 mM EDTA, 0.5 mM EGTA, 200 mM NaCl) and centrifuged again at 3724 *g* at 4°C. The pellet was then suspended in a nuclei lysis buffer (50 mM Tris-HCl, pH 8.1, 10 mM EDTA, 1% SDS) and incubated on ice for 10 min. Samples were sonicated using a Diagenode Bioruptor XL (30 sec on, 30 sec off for 30 min total) to generate chromatin fragments of between 200 and 400 bp.

Following sonication, samples were centrifuged for 10 min at 20000 *g* at 4°C. The supernatant was diluted and added to pre-incubated beads and antibody. Immunoprecipitation was performed using a mouse monoclonal antibody to RNA polymerase II (ab5408, Abcam) and a rabbit polyclonal antibody to Histone H3 (ab1791, Abcam) using Dynabeads Protein A (Invitrogen) according to the manufacturer's protocol. The unbound fraction was used for normalization. An IgG antibody (sc-2027, Santa Cruz Biotechnology) was used as a negative control. Samples were analyzed by western blot.

The immunoprecipitated samples were reverse crosslinked by adding 5 M NaCl overnight at 65°C. The samples were then treated with RNase A (Qiagen) for 30 min at 37°C followed by proteinase K (New England BioLabs) treatment for 4 hr at 65°C. DNA samples were purified using Promega Wizard SV Gel and PCR clean-up system, and amplified by QPCR as described above.

### RNA processivity analysis

After transfection of the minigene, nuclei were extracted as described above. The pellet was suspended in TRI reagent and nascent RNA was purified. QPCR analysis was performed with two pairs of primers: primer pair A amplified a region of intron 20 and primer pair B flanked the junction area between intron 20 and exon 21. Sequences will be provided upon request. cDNA was synthesized with the reverse primer of each pair. The ratio between the QPCR enrichments of B and A was calculated and the results were normalized to the 24 hr value.

## Supporting Information

Figure S1 **Constitutive splicing does not lead to reduced nucleosome occupancy as a function of time.** (**A**, **D**) The IKAP19–21 (**A**) or the IMP (**D**) minigene plasmids were cloned into a pEGFP-C3 vector (Clontech) and transfected into 293 cells. RNA samples were extracted 24, 48, or 72 hr following transfection. Splicing products were separated on a 2% agarose gel after RT-PCR reaction using appropriate primers. (**B**, **E**) The IKAP19–21 (**B**) or the IMP (**E**) minigenes were transfected into 293 cells. Pure nuclei were extracted 24 and 72 hr following transfection. Half of the nuclei were treated with MNase and half were untreated (input). Mononucleosomal DNA was extracted from an agarose gel and subjected to absolute QPCR analysis. Data was normalized to transfection efficiency using primers for the GFP area of the plasmid using input DNA. (**C**, **F**): IKAP19–21 (**C**) and IMP (**F**) minigenes were transfected into 293 cells. Cells were collected and used for ChIP experiment with H3 antibody and mouse IgG as a control. The precipitated DNA fragments were subjected to QPCR analysis. Enrichment values were normalized to the unbound fraction, to a non-specific IgG antibody, and to the GFP area of the plasmid. Results are presented as antibody fold change 72 hr compared to 24 hr following the transfection. The experiment was repeated independently twice. QPCR experiments were amplified in triplicate; results shown are mean values ± SD.
(TIF)

Figure S2 **The shift in alternative splicing is linked to chromatin organization.** (**A**) IKAP19–23 minigene was transfected into 293 cells. Cells were collected at 24 and 72 hr and used for an H3-ChIP analysis. The precipitated DNA fragments were subjected to QPCR. Enrichment values were normalized to the unbound fraction, to a non-specific IgG antibody, and to the GFP area of the plasmid. Results are presented as H3 fold change 72 hr compared to 24 hr following the transfection. All experiments were repeated independently three times, and the results shown are representative of an average experiment. QPCR experiments were amplified in triplicate; results shown are mean values ± SD. It should be noted that in the ChIP analysis it is not possible to compare histone binding to different exon and intron regions (a horizontal comparison) since relative results (fold change of 72 hr versus 24 hr) were measured in contrast to the absolute values presented in the MNase analysis. For the same reason, it is not possible to compare the values of ChIP data to MNase data. (**B**) The IKAP19–23 minigene was transfected into 293 cells. Nuclei were extracted 24 and 72 hr following transfection. Nuclei were digested with DNase I. Equal plasmid amounts were run on an agarose gel and subjected to Southern blot transfer and hybridized with DIG-GFP-labeled DNA probe.
(TIF)

Figure S3 **Chromatin organization affects alternative splicing**. (**A**) The IKAP19–23 minigene was transfected into 293 cells. TSA or NaB was added three hours after transfection at the indicated concentrations, and RNA was extracted 72 hr following the transfection. The splicing products were separated on a 2% agarose gel after RT-PCR reaction using appropriate primers. The PCR products were eluted and sequenced. (**B**) Similar to panel A except that CPT was added three hours after transfection at the indicated concentrations. RNA was extracted 24, 48, and 72 hr following transfection.
(TIF)

Figure S4 **Splicing pattern is altered by strengthening of the 5′ss.** (**A**) QPCR analysis of the level of the inclusion isoform (exon 19 through exon 23) compared to the skipped isoform (exon 19 and exon 23). The graph displays quantification of RT-PCR results presented in Figure 2A, lane 3 compared to lane 9. (**B**) Similar to panel A, only displaying quantitation of RT-PCR results presented in Figure 2C, lanes 3, 6, 9, and 12. Relative quantity represents normalization to the skipped isoform. QPCR experiments were amplified in triplicate; results shown are mean values ± SD.
(TIF)

Figure S5 **Alternative splicing affects nucleosome occupancy.** (**A**) Following transfection with the strong exon 20 5′ss minigene, cells were collected and used for an H3-ChIP analysis. The precipitated DNA fragments were subjected to QPCR. Enrichment values were normalized to the unbound fraction, to a non-specific IgG antibody and to the GFP area of the plasmid. Results are presented as H3 fold change between 72 and 24 hr samples. (**B**) Following co-transfection of IKAP19–23 and U1 plasmids, 72 hr after the transfection, H3-ChIP was performed as above. All experiments were repeated independently three times, and the results shown are representative of an average experiment. QPCR experiments were amplified in triplicate; results shown are mean values ± SD.
(TIF)

Figure S6 **SR proteins that alter the splicing pattern affect nucleosome occupancy.** (**A**) 293 cells were transfected with plasmids that express SRSF1, SRSF2, and hnRNPA1 and 24 hr later with the IKAP19–23 minigene. RNA was extracted 24 hr later and the splicing products were separated on a 2% agarose gel after RT-PCR. The PCR products were eluted and sequenced. (**B**) At 24 hr after the transfection with the minigene (48 hr after transfection of the SR plasmids), DNA was extracted from the nuclei of the cells. An MNase assay was then performed and the mononucleosomal DNA was subjected to absolute QPCR analysis on the alternative exon. Data are presented as DNA copy number and were normalized to transfection efficiency using primers for the GFP area of the plasmid using untreated samples.
(TIF)

Figure S7 **Inhibiting splicing affects nucleosome occupancy.** (**A**) The IKAP19–23 minigene was transfected into 293 cells. Cells were treated with 10 nM meayamycin three hours after transfection. RNA was extracted 24 hr following the transfection. The splicing products were separated on a 2% agarose gel after RT-PCR reaction using appropriate primers. (**B**) At 24 hr after the transfection and treatment with meayamycin, cells were collected and used for ChIP experiment with an H3 antibody. The precipitated DNA fragments were subjected to QPCR using primers that cover the exons of the minigene. Enrichment values were normalized to the unbound fraction, to a non-specific IgG antibody, and to the GFP area of the plasmid. Results are presented as H3 fold change of meayamycin-treated compared to control untreated cells.
(TIF)

Figure S8 **Transcription by RNA polymerase II is linked to splicing and chromatin organization.** (**A**) An RNAPII-ChIP was performed on cells transfected with the IKAP19–23 minigene 24 or 72 hr following the transfection. The precipitated DNA fragments were amplified by QPCR using primers that cover the exons of the minigene. Enrichment values were normalized to the unbound fraction, to a non-specific IgG antibody, and to the GFP area of the plasmid. Results are presented as RNAPII fold change between 72 and 24 hr. The experiment was repeated independently three times; results shown are mean values ± SD. (**B**) The IKAP19–23 minigene was transfected into 293 cells. Nuclei were extracted 24, 48, or 72 hr following transfection, and RNA was extracted. RNAPII processivity was determined by measuring the abundance of distal versus proximal pre-mRNAs in a given position, with the assumption that most pre-mRNA is a co-transcriptional intermediate, as described previously [2], [3]. The cDNAs were amplified from the nascent RNA with a different reverse primer for each reaction. QPCR analysis was performed using primers to two regions on the minigene (indicated as A and B), and the ratio between their enrichments was calculated. The results were normalized to the 24 hr value. The experiment was repeated independently twice; results shown are mean values ± SD. QPCR experiments were amplified in triplicate. (**C**) The IKAP19–23 minigene was transfected into 293 cells. DRB was added 3 hr after the transfection at the indicated concentrations, and RNA was extracted 72 hr following transfection. Splicing products were separated on a 2% agarose gel after RT-PCR using appropriate primers. (**D**) IKAP19–23 minigene was transfected into 293 cells and DRB was added to the medium. After 72 hr cells were collected and used for an H3-ChIP analysis. The precipitated DNA fragments were subjected to QPCR using primers that cover most of the minigene. Enrichment values were normalized to the unbound fraction, to a non-specific IgG antibody, and to the GFP area of the plasmid. Results are presented as H3 fold change in DRB-treated vs. untreated cells. ChIP experiments were repeated independently twice. QPCR experiments were amplified in triplicate; results shown are mean values ± SD.
(TIF)

Figure S9 **Inhibiting splicing affects RNAPII occupancy.** The IKAP19–23 minigene was transfected into 293 cells. At 24 hr after the transfection and treatment with meayamycin, cells were collected and used for ChIP experiment with an RNAPII antibody. The precipitated DNA fragments were subjected to QPCR using primers that cover the exons of the minigene. Enrichment values were normalized to the unbound fraction, to a non-specific IgG antibody, and to the GFP area of the plasmid. Results are presented as RNAPII fold change of meayamycin-treated compared to control untreated cells.
(TIF)

Figure S10 **Inhibiting splicing affects RNAPII occupancy of endogenous genes.** HeLa cells were treated with 10 nM meayamycin, and after 24 hr cells were collected and used for ChIP experiment with an RNAPII antibody. The precipitated DNA fragments were subjected to QPCR using primers that cover the exons of the minigene. Enrichment values were normalized to the unbound fraction and to a non-specific IgG antibody. Results are presented as RNAPII fold change of meayamycin-treated compared to control untreated cells.
(TIF)

Table S1 **Inclusion to skipping ratio of RT-PCR.**
(DOC)

References S1 **Supporting references.**
(DOCX)
