# Supplementary figures and images for: Pre-mRNA Splicing Is a Determinant of Nucleosome Organization
Source: PLoS One. 2013 Jan 10;8(1):e53506. doi: 10.1371/journal.pone.0053506 (PMC3542351; doi:10.1371/journal.pone.0053506)

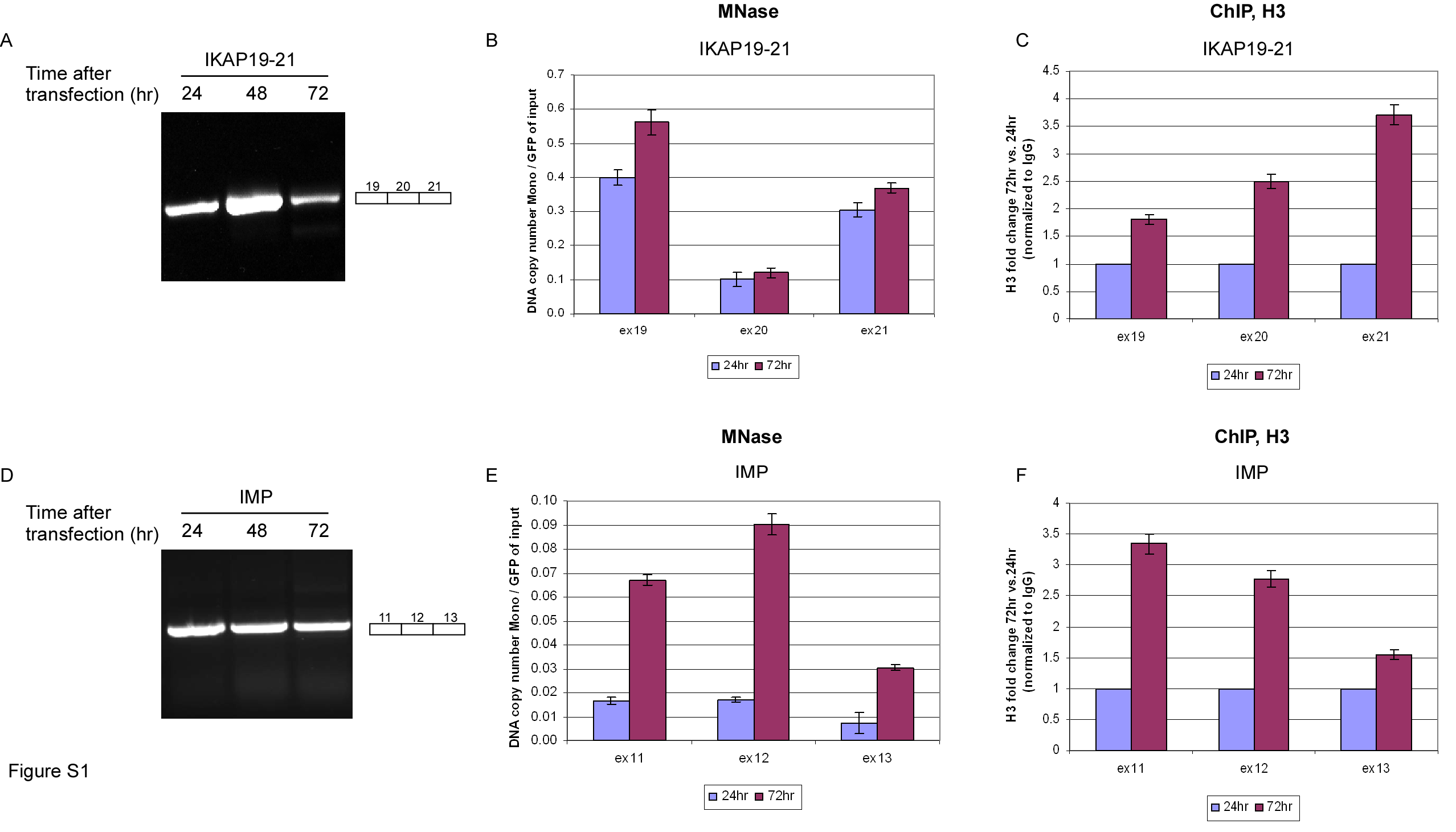

Supplement: Figure S1 — Constitutive splicing does not lead to reduced nucleosome occupancy as a function of time. (A, D) The IKAP19–21 (A) or the IMP (D) minigene plasmids were cloned into a pEGFP-C3 vector (Clontech) and transfected into 293 cells. RNA samples were extracted 24, 48, or 72 hr following transfection. Splicing products were separated on a 2% agarose gel after RT-PCR reaction using appropriate primers. (B, E) The IKAP19–21 (B) or the IMP (E) minigenes were transfected into 293 cells. Pure nuclei were extracted 24 and 72 hr following transfection. Half of the nuclei were treated with MNase and half were untreated (input). Mononucleosomal DNA was extracted from an agarose gel and subjected to absolute QPCR analysis. Data was normalized to transfection efficiency using primers for the GFP area of the plasmid using input DNA. (C, F): IKAP19–21 (C) and IMP (F) minigenes were transfected into 293 cells. Cells were collected and used for ChIP experiment with H3 antibody and mouse IgG as a control. The precipitated DNA fragments were subjected to QPCR analysis. Enrichment values were normalized to the unbound fraction, to a non-specific IgG antibody, and to the GFP area of the plasmid. Results are presented as antibody fold change 72 hr compared to 24 hr following the transfection. The experiment was repeated independently twice. QPCR experiments were amplified in triplicate; results shown are mean values ± SD. (TIF) [file pone.0053506.s001.tif]

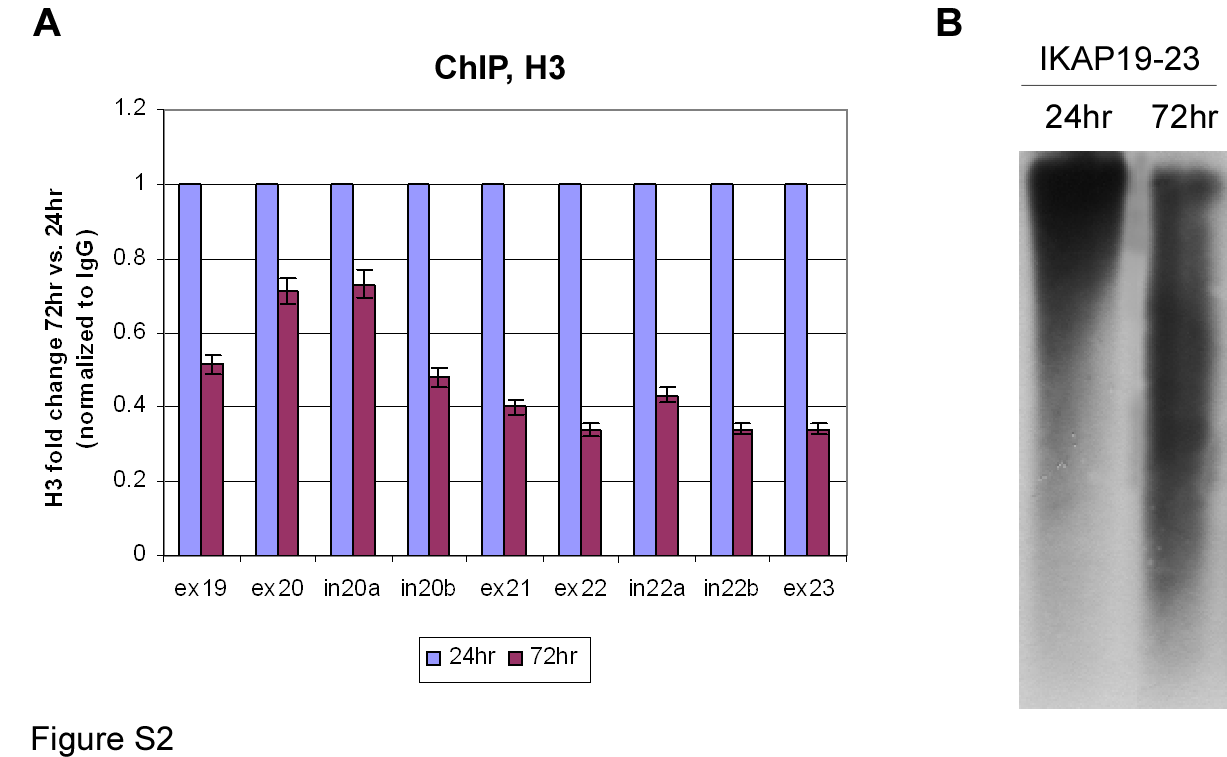

Supplement: Figure S2 — The shift in alternative splicing is linked to chromatin organization. (A) IKAP19–23 minigene was transfected into 293 cells. Cells were collected at 24 and 72 hr and used for an H3-ChIP analysis. The precipitated DNA fragments were subjected to QPCR. Enrichment values were normalized to the unbound fraction, to a non-specific IgG antibody, and to the GFP area of the plasmid. Results are presented as H3 fold change 72 hr compared to 24 hr following the transfection. All experiments were repeated independently three times, and the results shown are representative of an average experiment. QPCR experiments were amplified in triplicate; results shown are mean values ± SD. It should be noted that in the ChIP analysis it is not possible to compare histone binding to different exon and intron regions (a horizontal comparison) since relative results (fold change of 72 hr versus 24 hr) were measured in contrast to the absolute values presented in the MNase analysis. For the same reason, it is not possible to compare the values of ChIP data to MNase data. (B) The IKAP19–23 minigene was transfected into 293 cells. Nuclei were extracted 24 and 72 hr following transfection. Nuclei were digested with DNase I. Equal plasmid amounts were run on an agarose gel and subjected to Southern blot transfer and hybridized with DIG-GFP-labeled DNA probe. (TIF) [file pone.0053506.s002.tif]

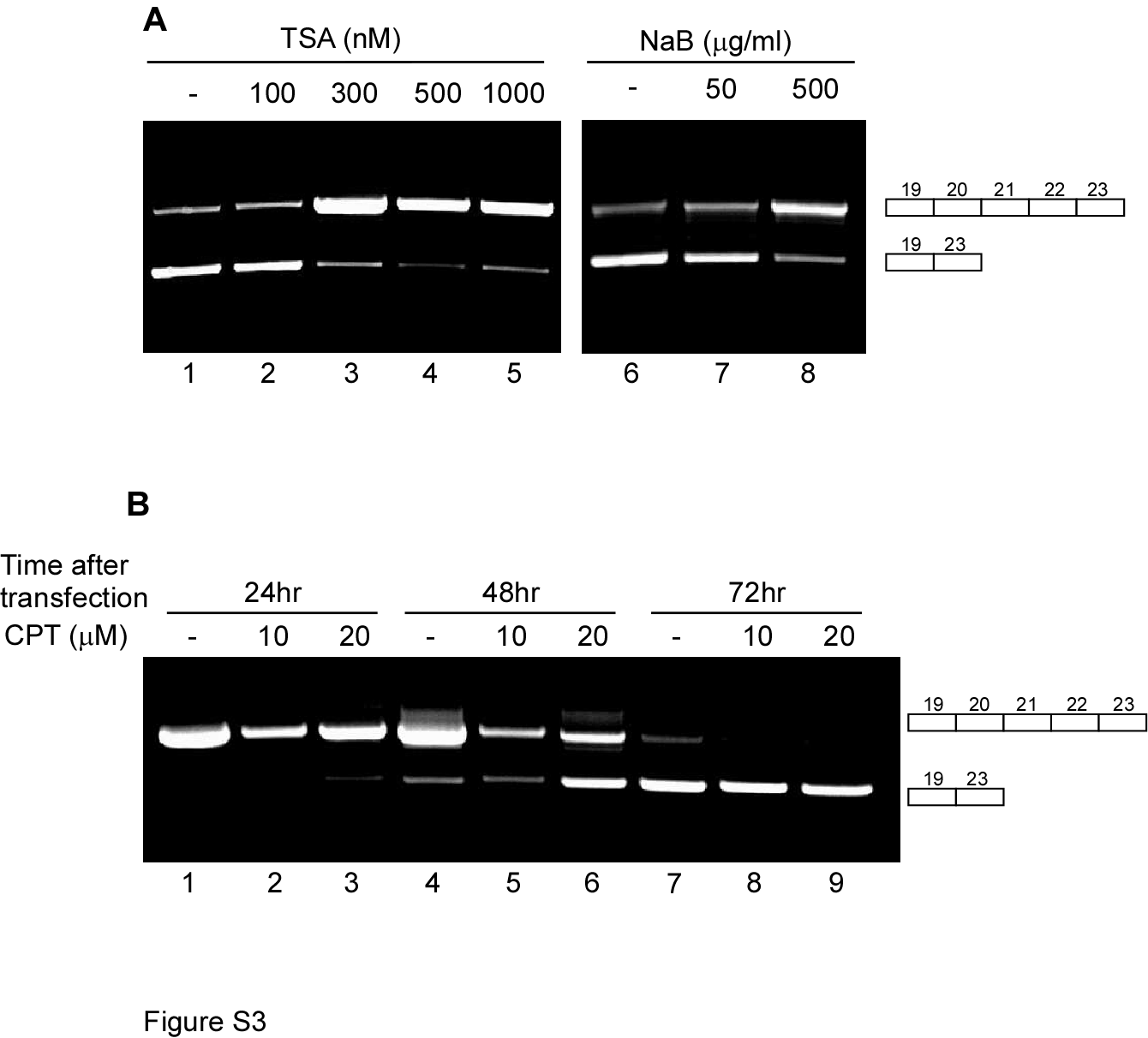

Supplement: Figure S3 — Chromatin organization affects alternative splicing. (A) The IKAP19–23 minigene was transfected into 293 cells. TSA or NaB was added three hours after transfection at the indicated concentrations, and RNA was extracted 72 hr following the transfection. The splicing products were separated on a 2% agarose gel after RT-PCR reaction using appropriate primers. The PCR products were eluted and sequenced. (B) Similar to panel A except that CPT was added three hours after transfection at the indicated concentrations. RNA was extracted 24, 48, and 72 hr following transfection. (TIF) [file pone.0053506.s003.tif]

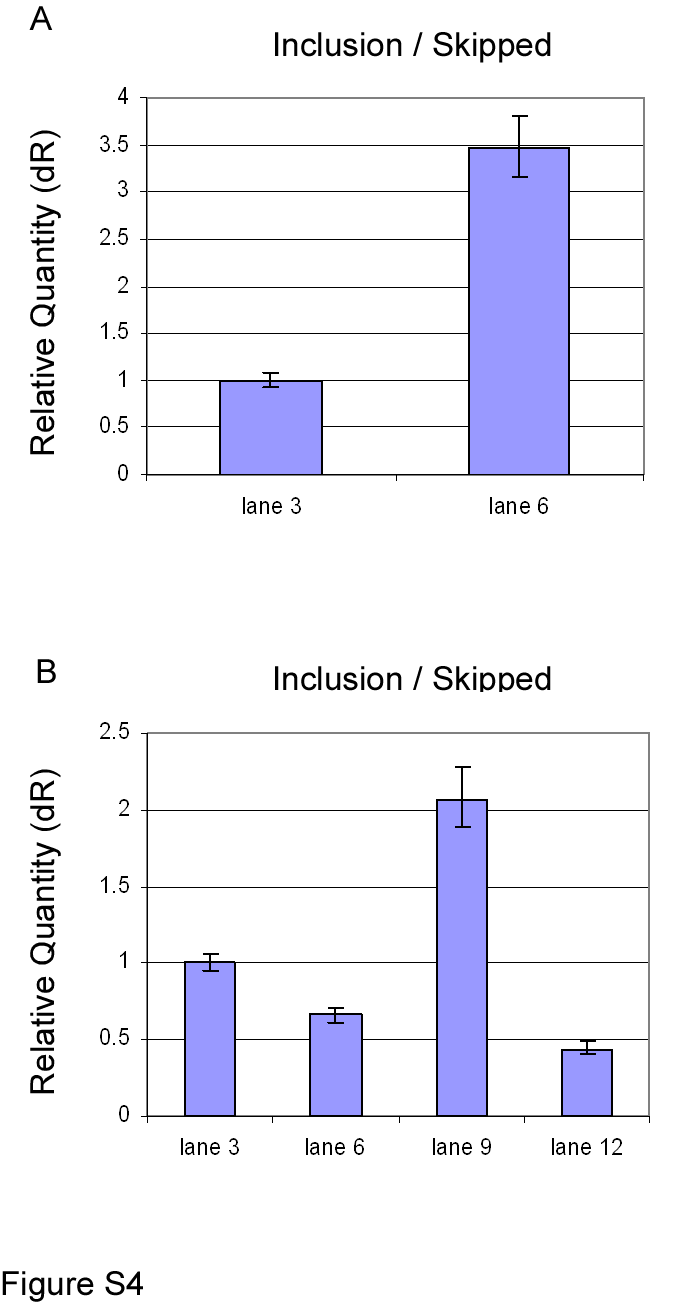

Supplement: Figure S4 — Splicing pattern is altered by strengthening of the 5′ss. (A) QPCR analysis of the level of the inclusion isoform (exon 19 through exon 23) compared to the skipped isoform (exon 19 and exon 23). The graph displays quantification of RT-PCR results presented in Figure 2A, lane 3 compared to lane 9. (B) Similar to panel A, only displaying quantitation of RT-PCR results presented in Figure 2C, lanes 3, 6, 9, and 12. Relative quantity represents normalization to the skipped isoform. QPCR experiments were amplified in triplicate; results shown are mean values ± SD. (TIF) [file pone.0053506.s004.tif]

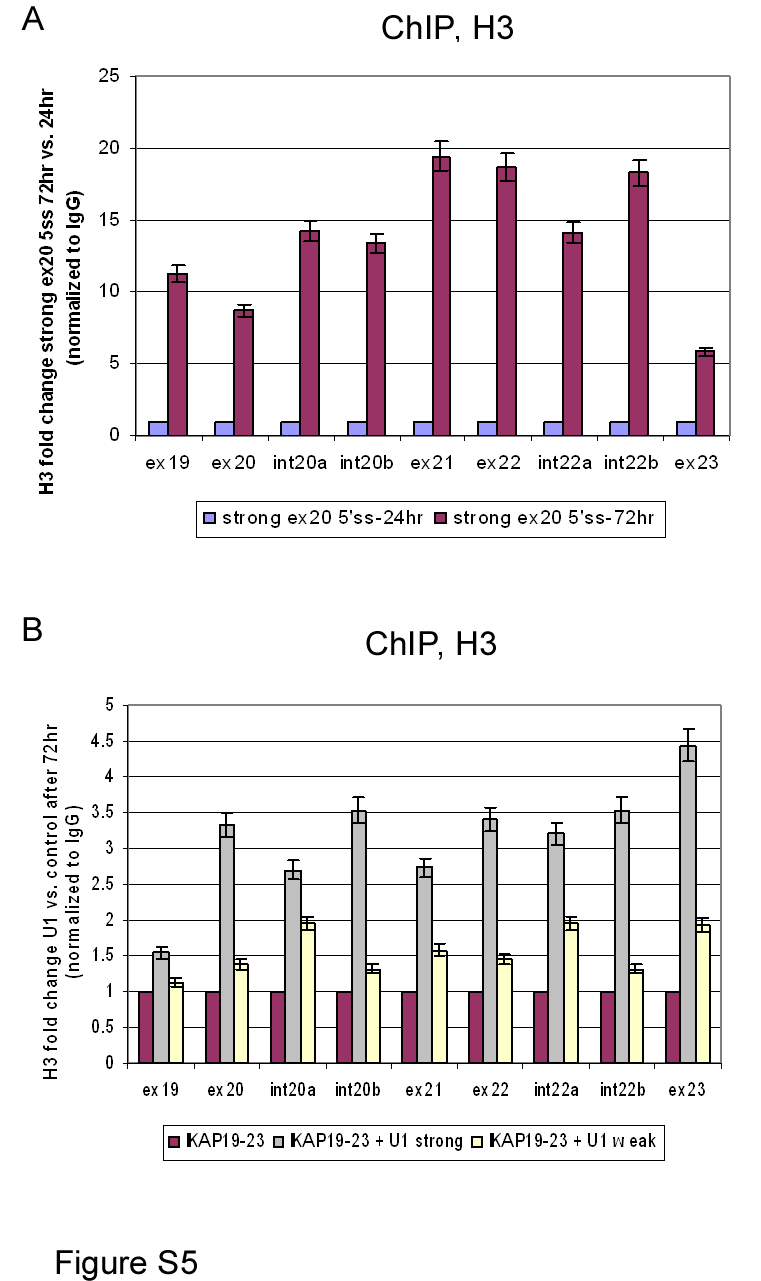

Supplement: Figure S5 — Alternative splicing affects nucleosome occupancy. (A) Following transfection with the strong exon 20 5′ss minigene, cells were collected and used for an H3-ChIP analysis. The precipitated DNA fragments were subjected to QPCR. Enrichment values were normalized to the unbound fraction, to a non-specific IgG antibody and to the GFP area of the plasmid. Results are presented as H3 fold change between 72 and 24 hr samples. (B) Following co-transfection of IKAP19–23 and U1 plasmids, 72 hr after the transfection, H3-ChIP was performed as above. All experiments were repeated independently three times, and the results shown are representative of an average experiment. QPCR experiments were amplified in triplicate; results shown are mean values ± SD. (TIF) [file pone.0053506.s005.tif]

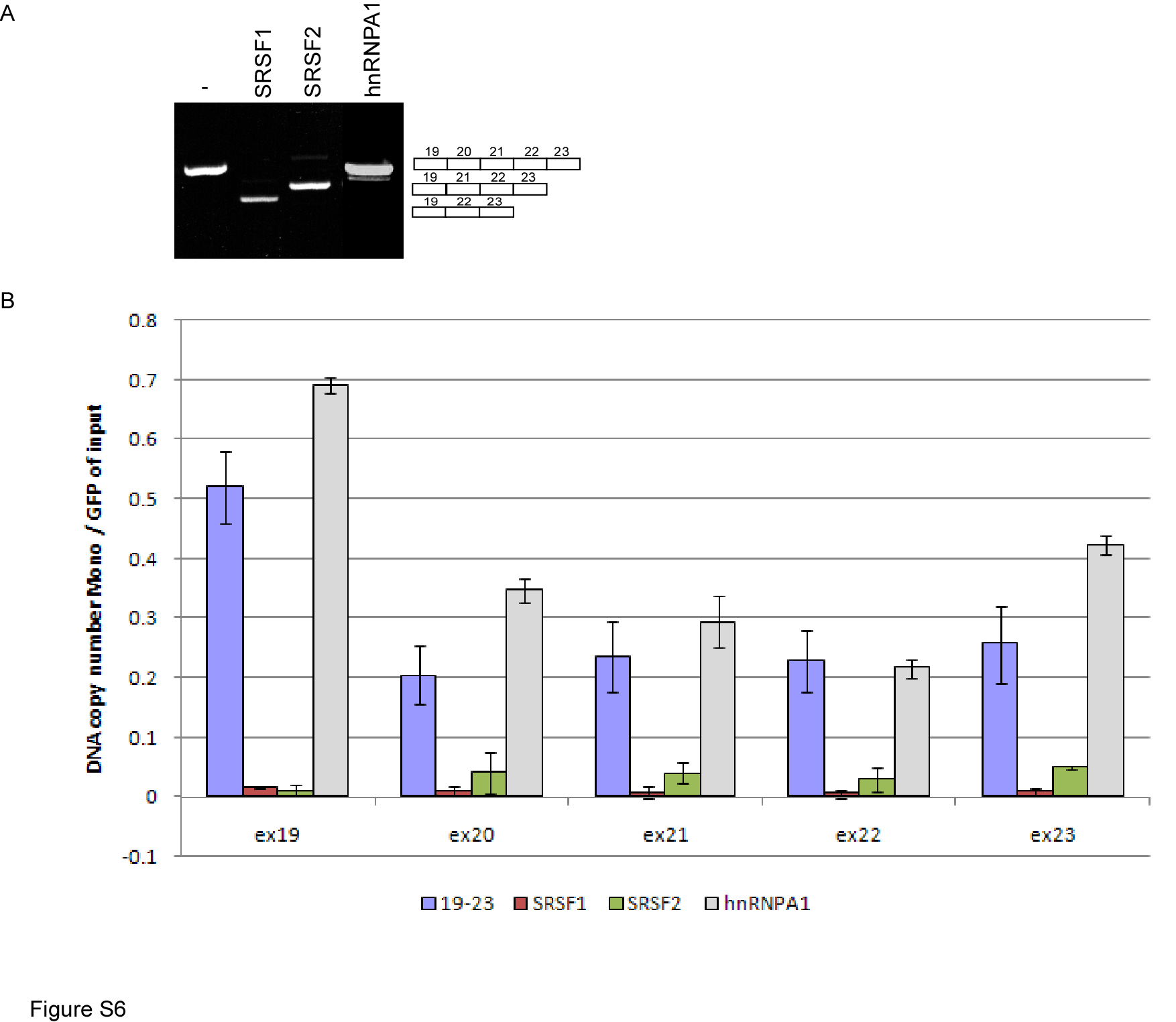

Supplement: Figure S6 — SR proteins that alter the splicing pattern affect nucleosome occupancy. (A) 293 cells were transfected with plasmids that express SRSF1, SRSF2, and hnRNPA1 and 24 hr later with the IKAP19–23 minigene. RNA was extracted 24 hr later and the splicing products were separated on a 2% agarose gel after RT-PCR. The PCR products were eluted and sequenced. (B) At 24 hr after the transfection with the minigene (48 hr after transfection of the SR plasmids), DNA was extracted from the nuclei of the cells. An MNase assay was then performed and the mononucleosomal DNA was subjected to absolute QPCR analysis on the alternative exon. Data are presented as DNA copy number and were normalized to transfection efficiency using primers for the GFP area of the plasmid using untreated samples. (TIF) [file pone.0053506.s006.tif]

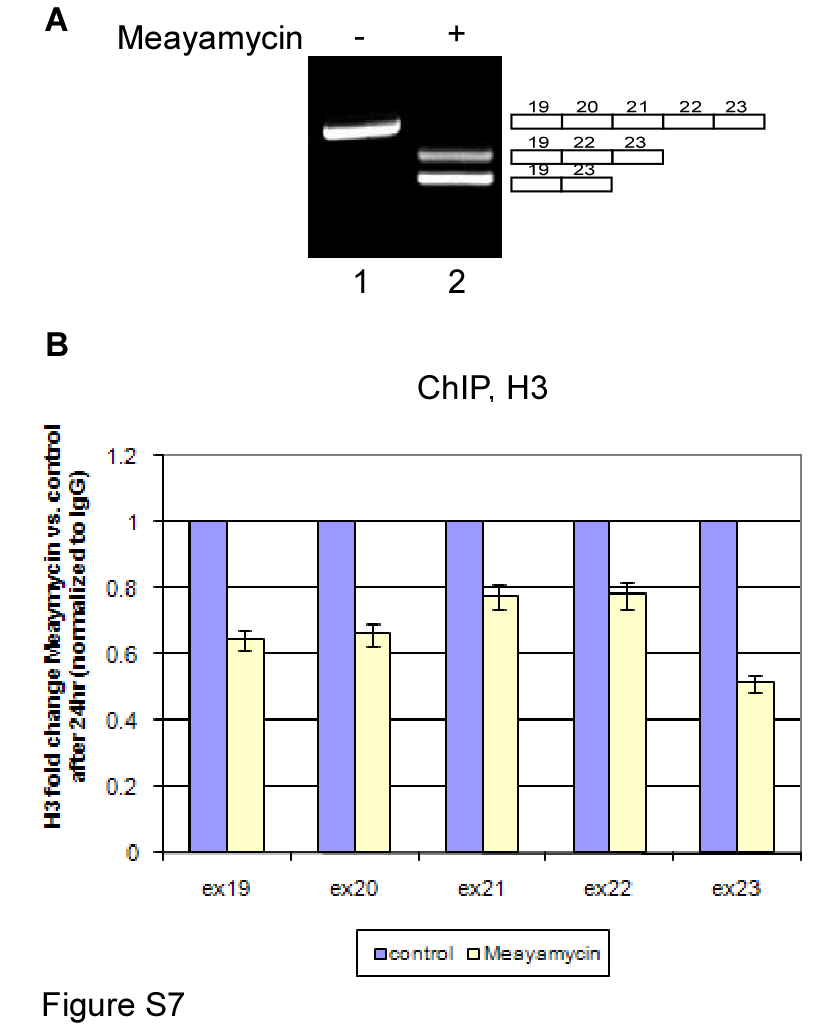

Supplement: Figure S7 — Inhibiting splicing affects nucleosome occupancy. (A) The IKAP19–23 minigene was transfected into 293 cells. Cells were treated with 10 nM meayamycin three hours after transfection. RNA was extracted 24 hr following the transfection. The splicing products were separated on a 2% agarose gel after RT-PCR reaction using appropriate primers. (B) At 24 hr after the transfection and treatment with meayamycin, cells were collected and used for ChIP experiment with an H3 antibody. The precipitated DNA fragments were subjected to QPCR using primers that cover the exons of the minigene. Enrichment values were normalized to the unbound fraction, to a non-specific IgG antibody, and to the GFP area of the plasmid. Results are presented as H3 fold change of meayamycin-treated compared to control untreated cells. (TIF) [file pone.0053506.s007.tif]

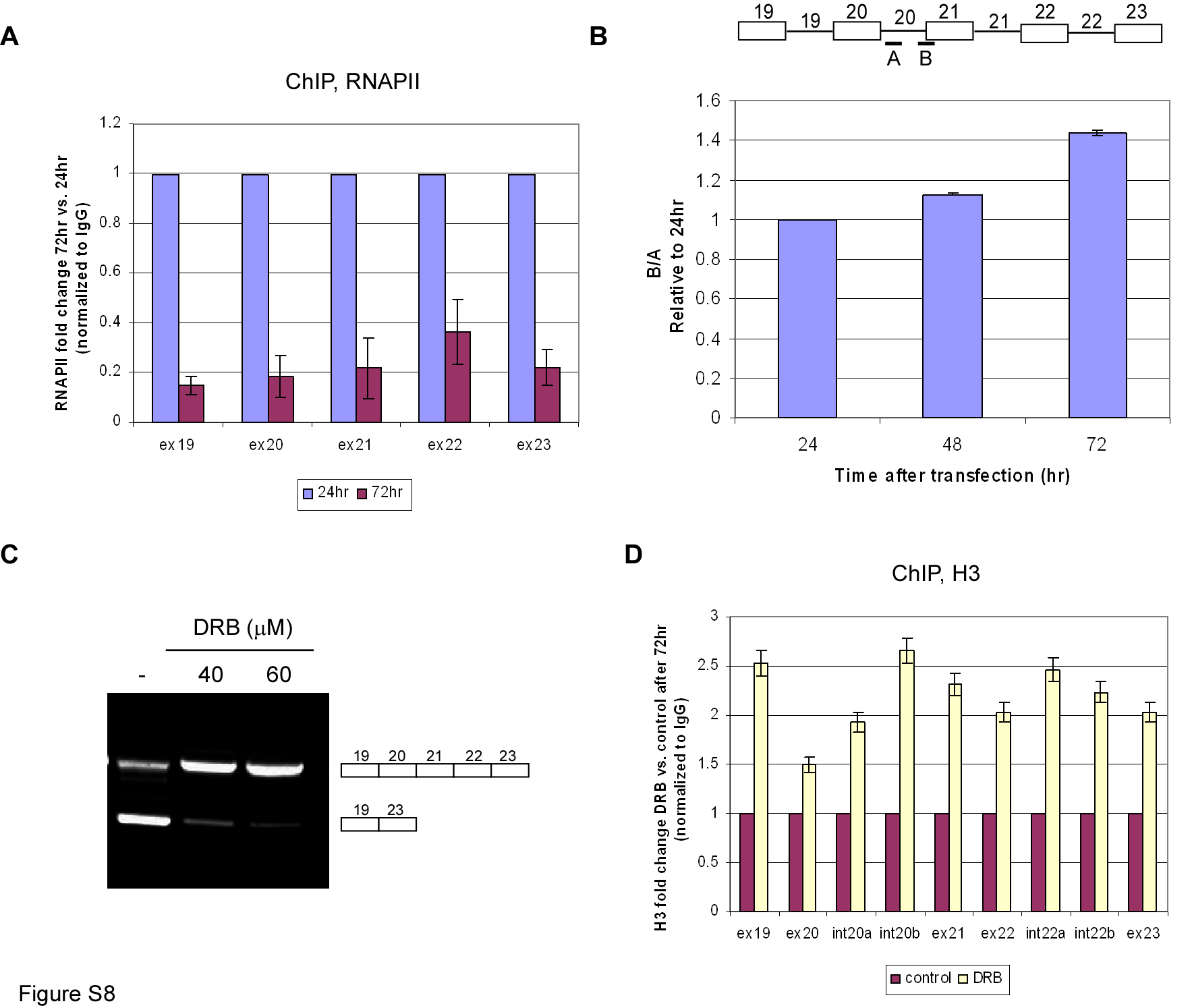

Supplement: Figure S8 — Transcription by RNA polymerase II is linked to splicing and chromatin organization. (A) An RNAPII-ChIP was performed on cells transfected with the IKAP19–23 minigene 24 or 72 hr following the transfection. The precipitated DNA fragments were amplified by QPCR using primers that cover the exons of the minigene. Enrichment values were normalized to the unbound fraction, to a non-specific IgG antibody, and to the GFP area of the plasmid. Results are presented as RNAPII fold change between 72 and 24 hr. The experiment was repeated independently three times; results shown are mean values ± SD. (B) The IKAP19–23 minigene was transfected into 293 cells. Nuclei were extracted 24, 48, or 72 hr following transfection, and RNA was extracted. RNAPII processivity was determined by measuring the abundance of distal versus proximal pre-mRNAs in a given position, with the assumption that most pre-mRNA is a co-transcriptional intermediate, as described previously [2], [3]. The cDNAs were amplified from the nascent RNA with a different reverse primer for each reaction. QPCR analysis was performed using primers to two regions on the minigene (indicated as A and B), and the ratio between their enrichments was calculated. The results were normalized to the 24 hr value. The experiment was repeated independently twice; results shown are mean values ± SD. QPCR experiments were amplified in triplicate. (C) The IKAP19–23 minigene was transfected into 293 cells. DRB was added 3 hr after the transfection at the indicated concentrations, and RNA was extracted 72 hr following transfection. Splicing products were separated on a 2% agarose gel after RT-PCR using appropriate primers. (D) IKAP19–23 minigene was transfected into 293 cells and DRB was added to the medium. After 72 hr cells were collected and used for an H3-ChIP analysis. The precipitated DNA fragments were subjected to QPCR using primers that cover most of the minigene. Enrichment values were normalized to the unbound fraction, to a [file pone.0053506.s008.tif]

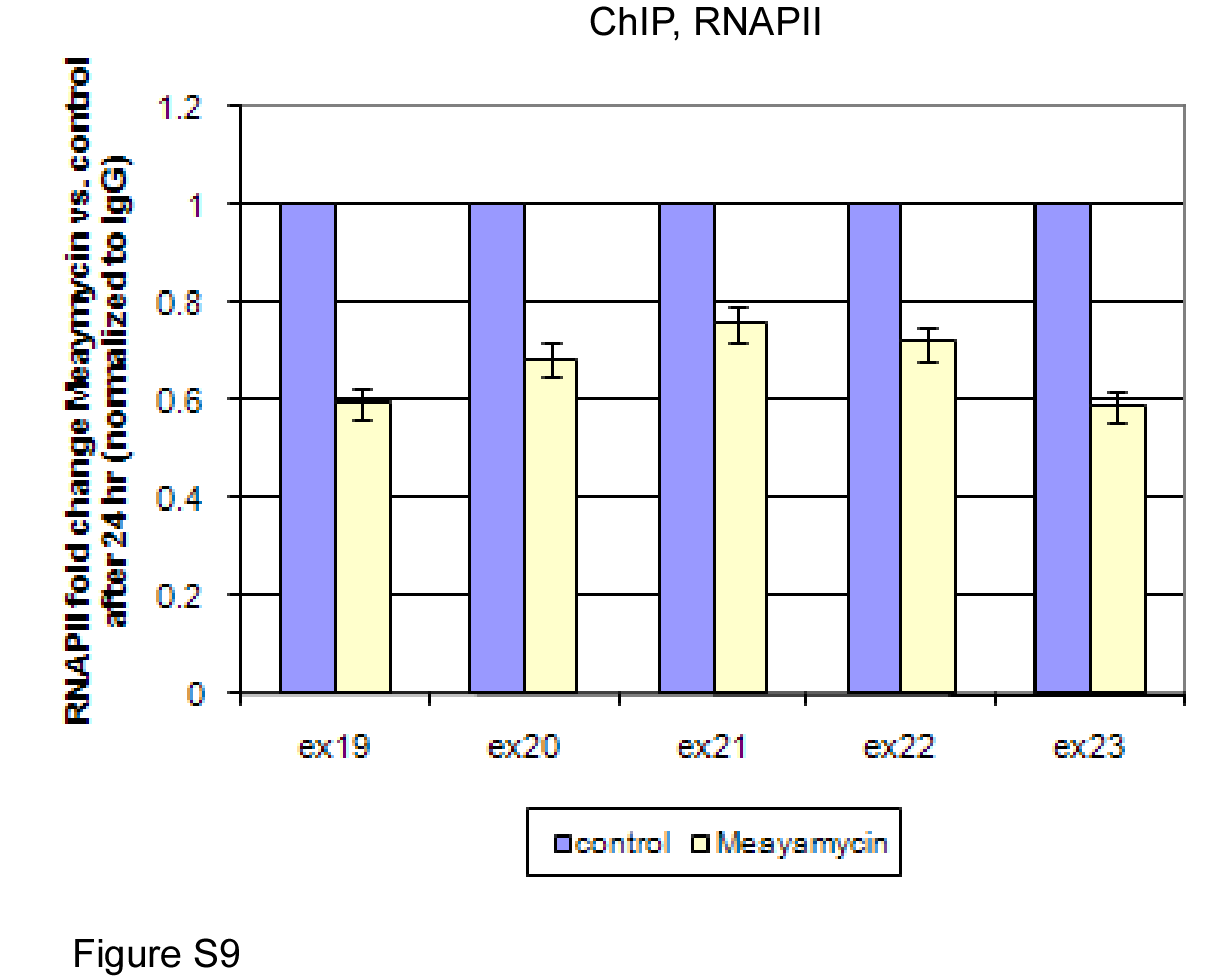

Supplement: Figure S9 — Inhibiting splicing affects RNAPII occupancy. The IKAP19–23 minigene was transfected into 293 cells. At 24 hr after the transfection and treatment with meayamycin, cells were collected and used for ChIP experiment with an RNAPII antibody. The precipitated DNA fragments were subjected to QPCR using primers that cover the exons of the minigene. Enrichment values were normalized to the unbound fraction, to a non-specific IgG antibody, and to the GFP area of the plasmid. Results are presented as RNAPII fold change of meayamycin-treated compared to control untreated cells. (TIF) [file pone.0053506.s009.tif]

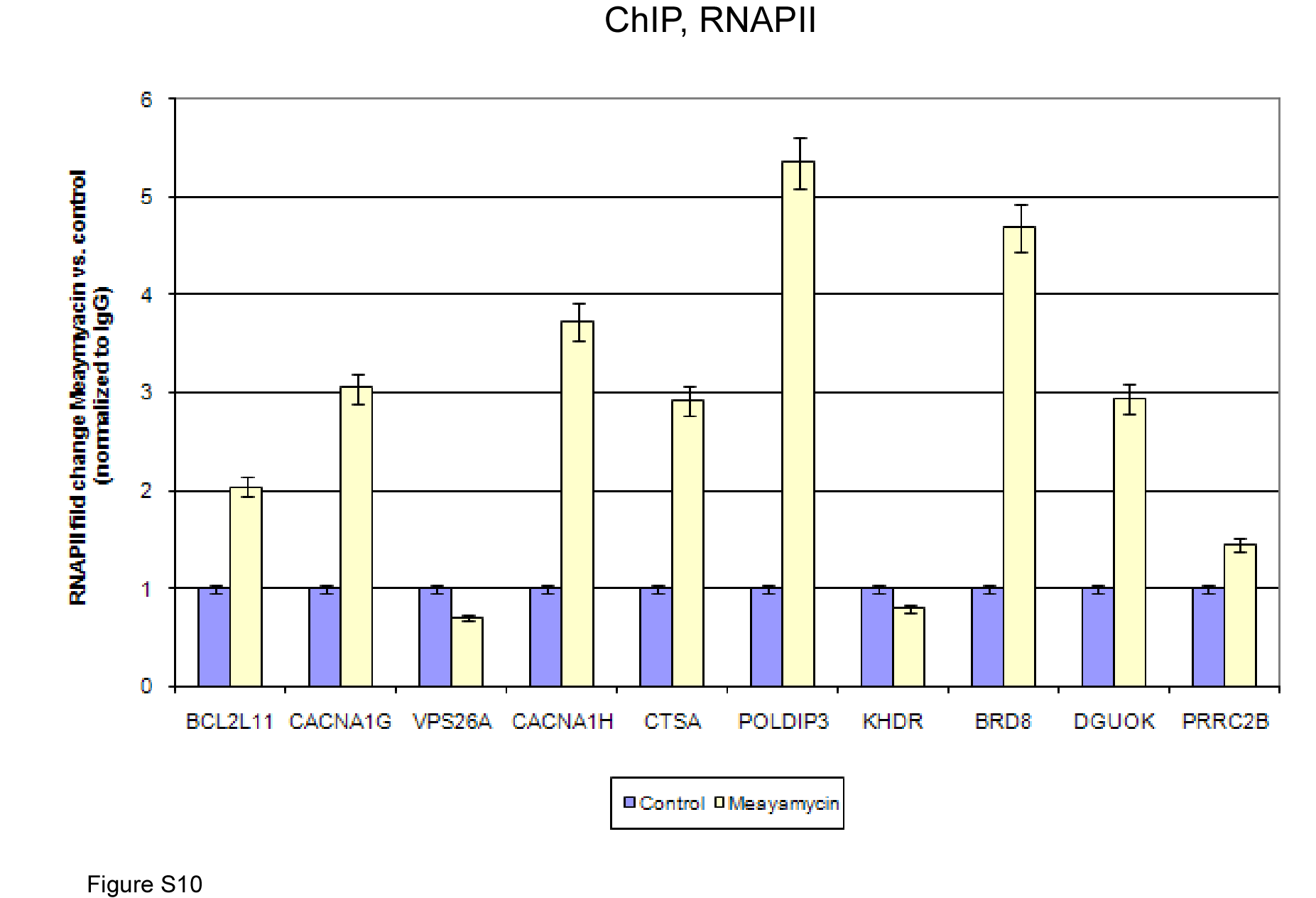

Supplement: Figure S10 — Inhibiting splicing affects RNAPII occupancy of endogenous genes. HeLa cells were treated with 10 nM meayamycin, and after 24 hr cells were collected and used for ChIP experiment with an RNAPII antibody. The precipitated DNA fragments were subjected to QPCR using primers that cover the exons of the minigene. Enrichment values were normalized to the unbound fraction and to a non-specific IgG antibody. Results are presented as RNAPII fold change of meayamycin-treated compared to control untreated cells. (TIF) [file pone.0053506.s010.tif]
