# Supplementary material for: Pre-mRNA Splicing Is a Determinant of Nucleosome Organization
Source: PLoS One. 2013 Jan 10;8(1):e53506. doi: 10.1371/journal.pone.0053506 (PMC3542351; doi:10.1371/journal.pone.0053506)
Supplement: Table S1 — Inclusion to skipping ratio of RT-PCR. (DOC) [file pone.0053506.s011.doc]

Table S1

Inclusion to skipping ratio.

| **Experiment** | **Inclusion to skipping ratio** |
| --- | --- |
| Fig. 1B, lane 1, IKAP19-23 24hr | 100 |
| Fig. 1B, lane 2, IKAP19-23 48hr | 84 |
| Fig. 1B, lane 3, IKAP19-23 72hr | 13 |
| Fig. 2A, lane 1, IKAP19-23 24hr | 100 |
| Fig. 2A, lane 2, IKAP19-23 48hr | 86 |
| Fig. 2A, lane 3, IKAP19-23 72hr | 11 |
| Fig. 2A, lane 4, ex20strong-ss 24hr | 100 |
| Fig. 2A, lane 5, ex20strong-ss 48hr | 100 |
| Fig. 2A, lane 6, ex20strong-ss 72hr | 95 |
| Fig. 2A, lane 7, ex21strong-ss 24hr | 100 |
| Fig. 2A, lane 8, ex21strong-ss 48hr | 100 |
| Fig. 2A, lane 9, ex21strong-ss 72hr | 65* |
| Fig. 2A, lane 10, ex22strong-ss 24hr | 100 |
| Fig. 2A, lane 11, ex22strong-ss 48hr | 100 |
| Fig. 2A, lane 12, ex22strong-ss 72hr | 64** |
| Fig. 2C, lane 1, IKAP19-23 24hr | 100 |
| Fig. 2C, lane 2, IKAP19-23 48hr | 93 |
| Fig. 2C, lane 3, IKAP19-23 72hr | 33 |
| Fig. 2C, lane 4, IKAP19-23+U1wt 24hr | 100 |
| Fig. 2C, lane 5, IKAP19-23+U1wt 48hr | 89 |
| Fig. 2C, lane 6, IKAP19-23+U1wt 72hr | 32 |
| Fig. 2C, lane 7, IKAP19-23+U1strong 24hr | 100 |
| Fig. 2C, lane 8, IKAP19-23+U1strong 48hr | 100 |
| Fig. 2C, lane 9, IKAP19-23+U1strong 72hr | 77 |
| Fig. 2C, lane 10, IKAP19-23+U1weak 24hr | 66** |
| Fig. 2C, lane 11, IKAP19-23+U1weak 48hr | 13** |
| Fig. 2C, lane 12, IKAP19-23+U1weak 72hr | 0 |
| Fig. S1A, lane 1, IKAP19-21 24hr | 100 |
| Fig. S1A, lane 2, IKAP19-21 48hr | 100 |
| Fig. S1A, lane 3, IKAP19-21 72hr | 100 |
| Fig. S1D, lane 1, IMP 24hr | 100 |
| Fig. S1D, lane 2, IMP 48hr | 100 |
| Fig. S1D, lane 3, IMP 72hr | 100 |
| Fig. S3A, lane 1, control | 26 |
| Fig. S3A, lane 2, TSA 100nM | 31 |
| Fig. S3A, lane 3, TSA 300nM | 88 |
| Fig. S3A, lane 4, TSA 500nM | 95 |
| Fig. S3A, lane 5, TSA 1000nM | 88 |
| Fig. S3A, lane 6, control | 25 |
| Fig. S3A, lane 7, NaB 50µg/ml | 38 |
| Fig. S3A, lane 8, NaB 500µg/ml | 78 |
| Fig. S3B, lane 1, 24hr control | 100 |
| Fig. S3B, lane 2, 24hr CPT 10µM | 100 |
| Fig. S3B, lane 3, 24hr CPT 20µM | 97 |
| Fig. S3B, lane 4, 48hr control | 92 |
| Fig. S3B, lane 5, 48hr CPT 10µM | 80 |
| Fig. S3B, lane 6, 48hr CPT 20µM | 51 |
| Fig. S3B, lane 7, 72hr control | 11 |
| Fig. S3B, lane 8, 72hr CPT 10µM | 0 |
| Fig. S3B, lane 9, 72hr CPT 20µM | 0 |
| Fig. S6A, lane 1, control | 100 |
| Fig. S6A, lane 2, SRSF1 | 0 |
| Fig. S6A, lane 3, SRSF2 | 0 |
| Fig. S6A, lane 2, hnRNPA1 | 100 |
| Fig. S7A, lane 1, control | 100 |
| Fig. S7A, lane 2, Meayamycin 10nM | 0 |
| Fig. S8C, lane 1, control | 33 |
| Fig. S8C, lane 2, DRB 40µM | 91 |
| Fig. S8C, lane 3, DRB 60µM | 97 |

* Fig. 2A, lane 9, ex21strong-ss 72hr– In the RT-PCR three bands are present: the inclusion isoform (exon 19 through exon 23), skipping of exon 20 and skipping of exon 20 and 21. No skipped isoform of the internal exons (exon 19-exon 23) is detected. The indicated splicing ratio refers to the ratio of the inclusion isoform compared to the other splicing isoforms.

** Fig. 2A, lane 12, ex22strong-ss 72hr, and Fig. 2C, lane 10, IKAP19-23+U1weak 24hr - In the RT-PCR three bands are present: the inclusion isoform (exon 19 through exon 23), skipping of exon 20 and skipping of the internal exons (exon 19-exon 23). For Fig. 2C, lane 11, IKAP19-23+U1weak 48hr, skipping of exon 21 is also detected. The indicated splicing ratio refers to the ratio of the inclusion isoform compared to the other splicing isoforms.
