# Supplementary material for: Pre-mRNA Splicing Is a Determinant of Nucleosome Organization
Source: PLoS One. 2013 Jan 10;8(1):e53506. doi: 10.1371/journal.pone.0053506 (PMC3542351; doi:10.1371/journal.pone.0053506)
Supplement: References S1 — Supporting references. (DOCX) [file pone.0053506.s012.docx]

SUPPLEMENTARY REFERENCES

1. Shapiro MB, Senapathy P (1987) RNA splice junctions of different classes of eukaryotes: sequence statistics and functional implications in gene expression. Nucleic Acids Res 15: 7155-7174.

2. de la Mata M, Alonso CR, Kadener S, Fededa JP, Blaustein M, et al. (2003) A slow RNA polymerase II affects alternative splicing in vivo. Mol Cell 12: 525-532.

3. Schor IE, Rascovan N, Pelisch F, Allo M, Kornblihtt AR (2009) Neuronal cell depolarization induces intragenic chromatin modifications affecting NCAM alternative splicing. Proc Natl Acad Sci U S A 106: 4325-4330.
